# MicroRNA-16 inhibits the TLR4/NF-κB pathway and maintains tight junction integrity in irritable bowel syndrome with diarrhea

**DOI:** 10.1016/j.jbc.2022.102461

**Published:** 2022-09-05

**Authors:** Meijuan Xi, Ping Zhao, Fang Li, Han Bao, Sijie Ding, Lijiang Ji, Jing Yan

**Affiliations:** 1 Digestive System Department, Changshu Hospital Affiliated to Nanjing University of Chinese Medicine, Changshu, China; 2 Department of Anorectal Surgery, Changshu Hospital Affiliated to Nanjing University of Chinese Medicine, Changshu, China; 3 First Clinical Medical College, Nanjing University of Chinese Medicine, Nanjing, China

**Keywords:** microRNA-16, toll-like receptor 4, NF-κB, X-inactive specific transcript, irritable bowel syndrome with diarrhea, AWR, abdominal withdrawal reflex, CCK-8, cell-counting kit-8, cDNA, complementary DNA, EMG, electromyography, FBS, fetal bovine serum, IBS, irritable bowel syndrome, IBS-D, IBS with diarrhea, LPS, lipopolysaccharide, NC, negative control, PDTC, pyrrolidine dithiocarbamate, qRT-PCR, quantitative RT-PCR, TER, transepithelial resistance, XIST, X-inactive specific transcript

## Abstract

Irritable bowel syndrome with diarrhea (IBS-D) is a chronic and relapsing inflammatory disorder in which pathogenesis has been shown to be in part the result of miRNA-mediated signaling. Here, we investigated the alleviatory role of miR-16 in IBS-D. First, we established an IBS-D mouse model using colonic instillation of acetic acid and developed an IBS-D cell model using lipopolysaccharide exposure. The experimental data demonstrated that miR-16 was underexpressed in the serum of IBS-D patients, as well as in the colorectal tissues of IBS-D mouse models and lipopolysaccharide-exposed intestinal epithelial cells. Next, miR-16 and TLR4 were overexpressed or inhibited to characterize their roles in the viability and apoptosis of intestinal epithelial cells, inflammation, and epithelial tight junction. We found that miR-16 overexpression increased the viability of intestinal epithelial cells, maintained tight junction integrity, and inhibited cell apoptosis and inflammation. We showed that miR-16 targeted TLR4 and inhibited the TLR4/NF-κB signaling pathway. Additionally, inhibition of NF-κB suppressed the long noncoding RNA XIST, thereby promoting enterocyte viability, inhibiting apoptosis and cytokine production, and maintaining tight junction integrity. *In vivo* experiments further verified the alleviatory effect of miR-16 on IBS-D symptoms in mice. Taken together, we conclude that miR-16 downregulates *XIST* through the TLR4/NF-κB pathway, thereby relieving IBS-D. This study suggests that miR-16 may represent a potential target for therapeutic intervention against IBS-D.

Irritable bowel syndrome (IBS) is a chronic and relapsing bowel disorder affecting 11% of the global population with notable disease burdens, for example, decreased productivity and reduced life quality (1). IBS is characterized by chronic abdominal discomfort and alternations in frequency and appearance of stool (2). Accordingly, IBS is further classified into three subtypes: IBS with diarrhea (IBS-D), IBS with constipation, and IBS with a mixed bowel pattern. Among these subtypes, IBS-D accounts for a quarter to a half of all IBS cases (1, 3).

The pathogenesis of IBS-D remains to be fully understood. Multiple etiological factors and triggers have been identified, including genetic susceptibility, visceral hypersensitivity, increased mucosal permeability, and altered gut microbiology (4). Emerging evidence has highlighted the significance of miRNAs as potential biomarkers against IBS (5). Martinez *et al.* (6) analyzed the differentially expressed miRNAs in IBS-D patients by RNA sequencing and identified miR-125b-5p and miR-16-5p as the most downregulated miRNAs in the context of IBS-D. The aforementioned findings encouraged us to undertake miRNA-involving IBS-D studies. miRNAs refer to short strands of RNA with the length of about 22 nucleotides that function as guide molecules to knock down the target mRNA and downregulate the corresponding proteins (7). Moreover, miRNAs were matured in the cytoplasm and can be transported by extracellular vehicles to the neighboring cells or into the circulatory system (8). Of note, miRNAs in the serum are recognized as potential targets for therapeutic agents for a variety of diseases. In this sense, we started an investigation from microRNA-16 (miR-16) in the serum of IBS-D patients and explored its downstream effectors.

*In silico* prediction of this study identified toll-like receptor 4 (TLR4) as a putative target of miR-16. Interestingly, TLR4 is a pathogen-recognition receptor of inflammation, which is contributory to IBS (9). In addition, intestinal barrier function could be recovered by wogonin through inactivating TLR4-dependent NF-κB pathway (10). Furthermore, NF-κB signaling pathway has been involved in diverse pathological responses, such as cancer and chronic inflammation, and has a role to confer in IBS (11, 12). Moreover, it has been documented that NF-κB signaling activated the expression of X-inactive specific transcript (XIST) (13), and XIST was positively related to the IBS (14). Thus, we hypothesized in the present study that miR-16 may present implications in biological processed of IBS-D, which may involve the TLR4/NF-κB/XIST axis.

## Results

### miR-16 is underexpressed in the IBS-D patients and mouse models

**Figure 1:**
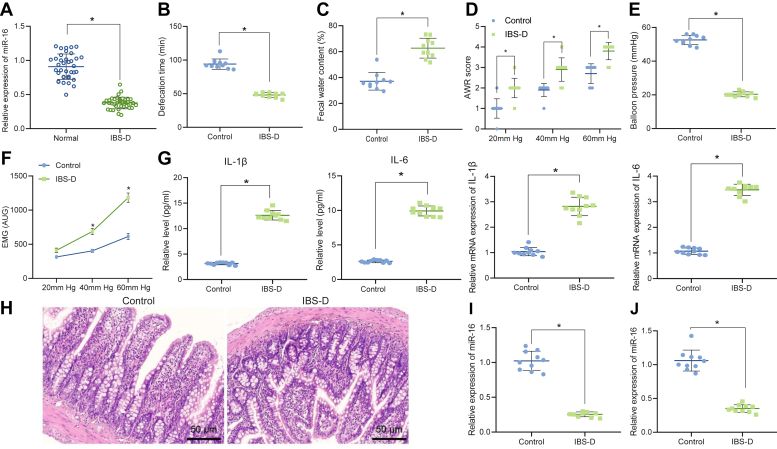
**miR-16 is underexpressed in IBS.***A*, qRT-PCR for determination of miR-16 in the serum of the control group and IBS patients. *B*, defecation intervals of control and IBS-D mice. *C*, stool types of control and IBS-D mice. *D*, AWR scores of control and IBS-D mice. *E*, detection of colorectal stepwise distention reaction in control and IBS-D mice. *F*, detection of EMG activity in control and IBS-D mice. *G*, ELISA and qRT-PCR for detection of IL-1β and IL-6 levels in the tissue homogenate of control and IBS-D mice. *H*, H&E staining detection of the pathological condition of rectal mucosa of control and IBS-D mice. *I*, qRT-PCR for determination of miR-16 in the control and IBS-D mouse colorectum. *J*, qRT-PCR for determination of miR-16 in control and IBS-D mouse serum. ∗*p* < 0.05. Measurement data were expressed as mean ± SD. Data comparison between the two groups was performed by unpaired *t* test. n = 10. IBS, irritable bowel syndrome; IBS-D, IBS with diarrhea; qRT-PCR, quantitative RT-PCR.

In order to reveal the role of miR-16 in IBS, we first measured the miR-16 expression in the serum from IBS-D patients. As shown in Figure 1*A*, miR-16 was underexpressed in the IBS-D patients. We then developed an IBS-D mouse model. IBS-D mice were depressive with decreased hair gloss and reduced food intake. Defecation intervals, stool types, and abdominal withdrawal reflex (AWR) of the mice were evaluated (Fig. 1, *B*–*D*). IBS-D mice presented with shorter defecation intervals, increased water content in stool, and higher AWR scores. Results of the colorectal stepwise distention (Fig. 1*E*) indicated that the injury threshold of IBS-D was decreased in comparison to the control mice. Electromyography (EMG) activity (Fig. 1*F*) in the IBS-D mice was enhanced *versus* the control mice.

As illustrated by ELISA and quantitative RT-PCR (qRT-PCR) (Fig. 1*G*), the cytokine production was elevated in the intestinal tissue homogenate of IBS-D mice, and levels of IL-1β and IL-6 were observed to be upregulated in IBS-D mice relative to the control mice. H&E staining assay was adopted to study the histologic change of the colon mucosa (Fig. 1*H*). No obvious inflammation or pathological changes were observed. These results indicated that the IBS-D mouse model was successfully established.

The level of miR-16 in the mouse colorectal tissue was measured by the qRT-PCR (Fig. 1*I*), and miR-16 was revealed to be poorly expressed in the IBS-D mice. Moreover, the serum level of miR-16 in IBS-D mice was confirmed to be downregulated as compared with that in control mice (Fig. 1*J*).

Taken together, our data demonstrated the downregulation of miR-16 in both IBS-D patients and mice.

### miR-16 overexpression promotes enterocyte viability, inhibits apoptosis and cytokine production, and maintains tight junction integrity

**Figure 2:**
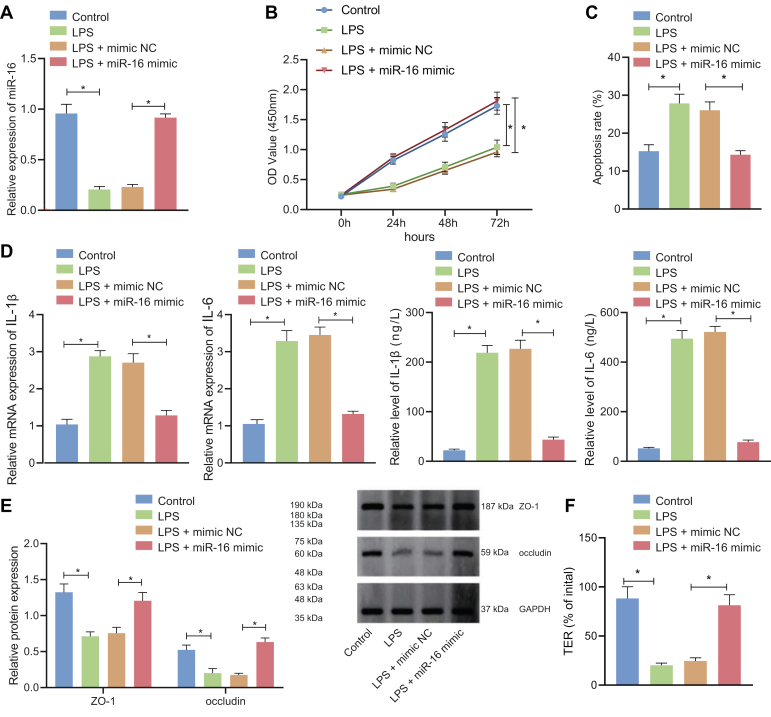
**miR-16 promotes enterocyte viability, maintains tight junction integrity, and inhibits apoptosis and cytokine production.***A*, qRT-PCR for determination of miR-16 level in LPS-induced NCM460 cells treated with miR-16 mimic. *B*, CCK-8 detection of LPS-induced NCM460 cell viability following treatment with miR-16 mimic. *C*, flow cytometry of LPS-induced NCM460 cell apoptosis following treatment with miR-16 mimic. *D*, ELISA and qRT-PCR detection of IL-1β and IL-6 levels in the LPS-induced NCM460 cell culture medium following treatment with miR-16 mimic. *E*, Western blot detection of expression of tight junction proteins ZO-1 and occludin in LPS-induced NCM460 cells treated with miR-16 mimic. *F*, TER was determined to reflect the tight junction integrity in the LPS-induced NCM460 cells treated with miR-16 mimic. ∗*p* < 0.05. Measurement data were expressed as mean ± SD. Data comparison between the two groups was performed by unpaired *t* test. Statistical analysis concerning time-based measurements within each group was realized using repeated measures ANOVA with Bonferroni’s post hoc test. All cell experiments were conducted independently in triplicate. CCK-8, cell-counting kit-8; LPS, lipopolysaccharide; qRT-PCR, quantitative RT-PCR; TER, transepithelial resistance.

To investigate the effect of miR-16 on the intestinal epithelial cells, we established the lipopolysaccharide (LPS)-induced IBS-D model in the normal colonic epithelial cell line NCM460 (15), where miR-16 was subsequently overexpressed. Relative to the control group, miR-16 expression in the LPS-treated intestinal epithelial cells was remarkably decreased. Relative to the LPS + mimic negative control (NC) group, miR-16 mimic upregulated the miR-16 expression (Fig. 2*A*).

Cell-counting kit-8 (CCK-8) assay and flow cytometry (Fig. 2, *B* and *C*) illustrated that cell viability in the LPS-induced intestinal epithelial cells was impeded and the apoptosis was accelerated *versus* the control cells, which could then be reversed in response to miR-16 mimic transfection. As revealed by ELISA and qRT-PCR (Fig. 2*D*), expression of IL-1β and IL-6 was enhanced in the LPS-treated intestinal epithelial cells. Whereas, miR-16 mimic suppressed IL-1β and IL-6 levels. Epithelial tight junction proteins ZO-1 and occludin were downregulated in the IBS-D cell model, and their levels were restored after the cells were treated with miR-16 mimic, as verified by Western blot (Fig. 2*E*).

Further, the tight junction integrity was compromised by LPS treatment, as reflected by reduced transepithelial resistance (TER) level, and additional miR-16 mimic transfection led to restored TER level, indicating enhanced tight junction integrity (Fig. 2*F*).

These data supported the finding that overexpression of miR-16 promoted enterocyte viability, inhibited apoptosis and cytokine production, and maintained tight junction integrity.

### miR-16 promotes enterocyte viability, inhibits apoptosis and cytokine production, and maintains tight junction integrity by inhibiting TLR4/NF-κB signaling pathway

**Figure 3:**
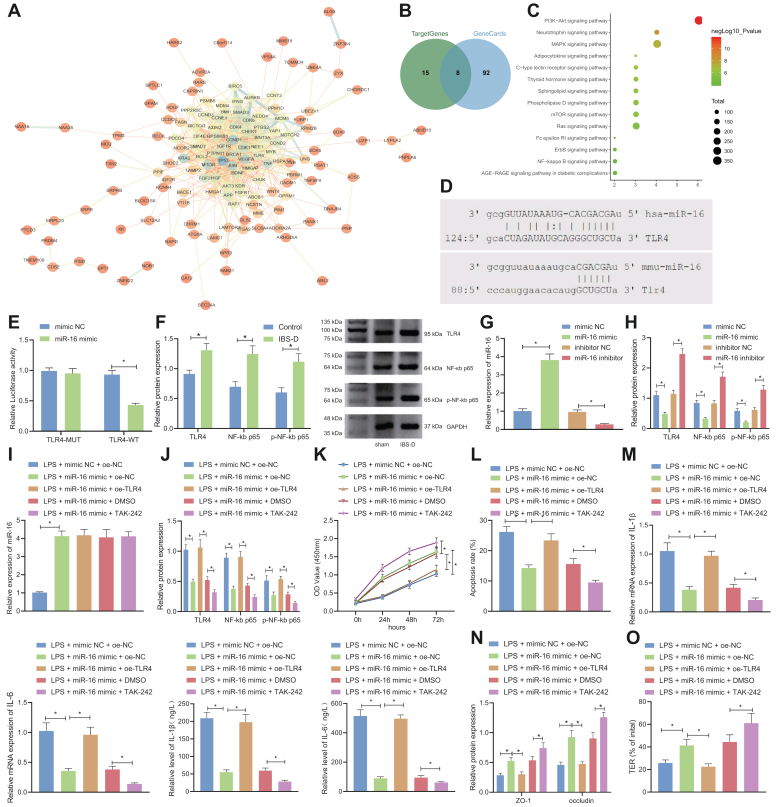
**miR-16 promotes enterocyte viability, maintains tight junction integrity, and inhibits apoptosis and cytokine production by inhibiting the TLR4/NF-κB signaling pathway.***A*, interaction of 161 genes analyzed by STRING and visualized by Cytoscape 3.5.1. *Blue node* indicates a high degree while *orange* a low degree. A thick link between the nodes indicates a close coexpression relation while a thin link indicates a far relation. *B*, Venn diagram of the miR-16 target gene and IBS target genes. *C*, plot of KEGG enrichment. The abscissa represents the number of genes involved in the pathway and the ordinate represents the name of the pathway. The color of the *dots* indicates −log10 *p*-value. *D*, the binding site of miR-16 with TLR4 in mouse and human predicted by microRNA.org. *E*, the binding of miR-16 to TLR4 in HEK293T cells verified by dual-luciferase reporter assay. *F*, Western blot detection of TLR4, NF-κB, and p65p-NF-κB p65 levels in the colorectal tissue of control and IBS-D mice; *G*, qRT-PCR determination of miR-16 expression in LPS-induced NCM460 cells treated with miR-16 mimic or inhibitor; *H*, Western blot detection of TLR4, NF-κB p65, and p-NF-κB p65 expression in LPS-induced NCM460 cells treated with miR-16 mimic or inhibitor. *I*, qRT-PCR for determination of miR-16 in LPS-induced NCM460 cells treated with miR-16 mimic alone or combined with oe-TLR4/TAK-242. *J*, Western blot detection of TLR4, NF-κB p65, and p-NF-κB p65 expression in LPS-induced NCM460 cells treated with miR-16 mimic alone or combined with oe-TLR4/TAK-242. *K*, CCK-8 assay of LPS-induced NCM460 cell viability following treatment with miR-16 mimic alone or combined with oe-TLR4/TAK-242. *L*, flow cytometry of LPS-induced NCM460 cell apoptosis following treatment with miR-16 mimic alone or combined with oe-TLR4/TAK-242. *M*, ELISA and qRT-PCR for detection of IL-1β and IL-6 levels in the culture medium of LPS-induced NCM460 cells treated with miR-16 mimic alone or combined with oe-TLR4/TAK-242. *N*, Western blot detection of expression of tight junction proteins ZO-1 and occludin in LPS-induced NCM460 cells treated with miR-16 mimic alone or combined with oe-TLR4/TAK-242. *O*, TER was determined to reflect the tight junction integrity in the LPS-induced NCM460 cells treated with miR-16 mimic alone or combined with oe-TLR4/TAK-242. ∗*p* < 0.05. Measurement data were expressed as mean ± SD. Comparison of data among multiple groups was performed by one-way ANOVA, followed by Tukey’s post hoc test. Statistical analysis concerning time-based measurements within each group was realized using repeated measures ANOVA with Bonferroni’s post hoc test. All cell experiments were conducted independently in triplicate. IBS, irritable bowel syndrome; IBS-D, IBS with diarrhea; CCK-8, cell-counting kit-8; LPS, lipopolysaccharide; qRT-PCR, quantitative RT-PCR; TER, transepithelial resistance.

The downstream modulation factors of miR-16 were predicted by RNAInter (score > 0.8). The interaction of the 161 downstream genes was analyzed by STRING and visualized by Cytoscape 3.7.2 software package (https://cytoscape.org/), as illustrated in Figure 3*A*. There were 23 genes in the core position of the network (degree > 20). Searching in GeneCards yielded 100 IBS-related genes. Intersection of the 23 core genes and the 100 IBS-related genes revealed eight candidate genes, namely TP53, KRAS, MTOR, TNF, BRCA1, BDNF, PTPN11, and TLR4 (Fig. 3*B*).

To further understand the modulation pathway, we analyzed the KEGG pathway involving the eight candidate genes using KOBAS (KEGG orthology-based annotation system, Fig. 3*C*). The relationship between TLR4 and IBS was meanwhile corroborated by previous literature (10). The binding site of miR-16 and TLR4 was unveiled from the microRNA.org (Fig. 3*D*) and confirmed by dual-luciferase reporter assay (Fig. 3*E*). TLR4-WT and miR-16 mimic cotransfection reduced the luciferase activity, while no significant difference was observed in luciferase activity between TLR4 MUT and miR-16 mimic cotransfection group and the mimic NC group. Therefore, miR-16 specifically binds to TLR4 mRNA on the translational level.

The expression of TLR4, NF-κB p65, phosphorylated (p)-NF-κB p65 in the colorectal tissue of the IBS-D model was determined by Western blot (Fig. 3*F*), which were observed to be highly expressed in the IBS-D model. Further, the expression of miR-16, TLR4, NF-κB p65, and p-NF-κB p65 was measured by qRT-PCR and Western blot in response to mimic or inhibitor of miR-16 in the human normal colonic epithelial cell line NCM460 (Fig. 3, *G* and *H*). miR-16 was upregulated, while TLR4, NF-κB p65, and p-NF-κB p65 were downregulated in the presence of miR-16 mimic. In contrast, miR-16 inhibitor led to the opposite results. Thus, we confirmed that miR-16 inhibited the TLR4/NF-κB signaling pathway.

Overexpression of miR-16, co-overexpression of miR-16 and TLR4, and overexpression of miR-16 with TLR4 inhibitor TAK-242 simultaneously were applied on the LPS-treated NCM460 cells. The results of qRT-PCR and Western blot (Fig. 3, *I* and *J*) suggested that miR-16 was elevated in the LPS-treated NCM460 cells transfected with miR-16 mimic while expression of TLR4, NF-κB p65, and p-NF-κB p65 was attenuated. Overexpression of TLR4 elevated the levels of TLR4, NF-κB p65, and p-NF-κB p65 in LPS-treated NCM460 cells transfected with miR-16 mimic. The addition of TAK-242 and miR-16 mimic presented higher levels of TLR4, NF-κB p65, and p-NF-κB p65 in LPS-treated NCM460 cells, as compared with miR-16 mimic alone.

CCK-8 assay and flow cytometry (Fig. 3, *K* and *L*) indicated that transfection of miR-16 mimic augmented the cell viability and impeded the apoptosis, while further overexpression of TLR4 reversed the results. Relative to miR-16 mimic alone, simultaneous treatment of TAK-242 and miR-16 mimic promoted the NCM460 cell viability and repressed the apoptosis. Inflammatory cytokines were monitored by ELISA, qRT-PCR, and Western blot (Fig. 3, *M* and *N*). miR-16 mimic repressed the IL-1β and IL-6 and raised the level of ZO-1 and occludin, which could then be reversed by co-overexpression of TLR4. TAK-242 treatment in the LPS-treated NCM460 cells overexpressing miR-16 further repressed the IL-1β and IL-6 expression and elevated the level of ZO-1 and occludin. Subsequent TER detection indicated that the LPS-induced damage to tight junction integrity was relieved in response to miR-16 mimic, and the effect of miR-16 mimic alone was negated by additional TLR4 overexpression but potentiated when miR-16 mimic was combined with TAK-242 treatment (Fig. 3*O*).

Together, miR-16 inhibited the TLR4/NF-κB signaling pathway to augment enterocyte viability, to inhibit apoptosis and cytokine production, and to maintain tight junction integrity.

### Inhibition of NF-κB suppresses XIST to augment enterocyte viability, inhibits apoptosis and cytokine production, and maintains tight junction integrity

**Figure 4:**
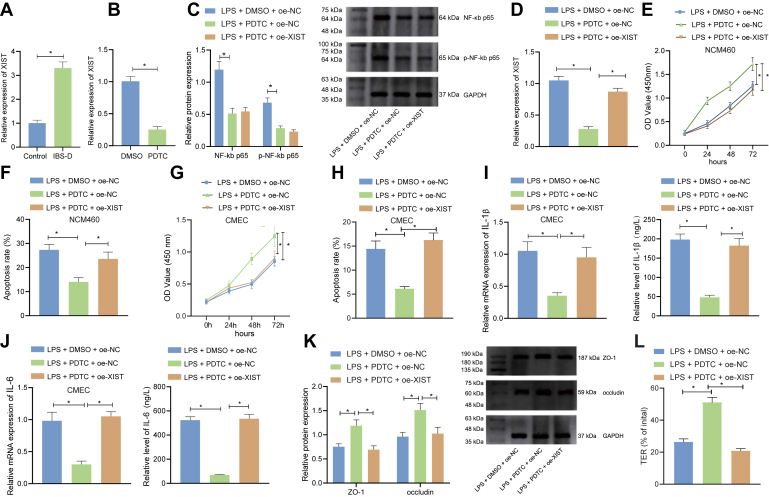
**Inhibition of NF-κB suppresses XIST to augment enterocyte viability, maintain tight junction integrity, and inhibit apoptosis and cytokine production.***A*, qRT-PCR for determination of XIST level in the colorectal tissue of control and IBS-D mice. *B*, qRT-PCR for determination of XIST level in LPS-induced NCM460 cells treated with PDTC for 1 h. *C*, Western blot detection of NF-κB p65 and p-NF-κB p65 levels in LPS-induced NCM460 cells treated with PDTC for 1 h alone or combined with oe-XIST. *D*, qRT-PCR for determination of XIST level in LPS-induced NCM460 cells treated with PDTC for 1 h alone or combined with oe-XIST. *E*, CCK-8 assay of LPS-induced NCM460 cell viability following treatment with PDTC for 1 h alone or combined with oe-XIST. *F*, flow cytometry of LPS-induced NCM460 cell apoptosis following treatment with PDTC for 1 h alone or combined with oe-XIST. *G*, CCK-8 assay of LPS-induced CMEC cell viability following treatment with PDTC for 1 h alone or combined with oe-XIST. *H*, flow cytometry of LPS-induced CMEC cell apoptosis following treatment with PDTC for 1 h alone or combined with oe-XIST. *I* and *J*, ELISA and qRT-PCR for detection of IL-1β (*I*) and IL-6 (*J*) level in the culture medium of LPS-induced CMEC cells treated with PDTC for 1 h alone or combined with oe-XIST. *K*, Western blot detection of expression of tight junction proteins ZO-1 and occludin in LPS-induced CMEC cells treated with PDTC for 1 h alone or combined with oe-XIST. *L*, TER was determined to reflect the tight junction integrity in LPS-induced CMEC cells treated with PDTC for 1 h alone or combined with oe-XIST. ∗*p* < 0.05. Measurement data were expressed as mean ± SD. Data comparison between two groups was performed by unpaired *t* test. Comparison of data among multiple groups was performed by one-way ANOVA, followed by Tukey’s post hoc test. Statistical analysis concerning time-based measurements within each group was realized using repeated measures ANOVA with Bonferroni’s post hoc test. All cell experiments were conducted independently in triplicate. CCK-8, cell-counting kit-8; LPS, lipopolysaccharide; PDTC, pyrrolidine dithiocarbamate; qRT-PCR, quantitative RT-PCR; TER, transepithelial resistance; XIST, X-inactive specific transcript.

We examined the XIST expression in the colorectal tissue of the IBS-D model using qRT-PCR (Fig. 4*A*), which revealed that XIST was highly expressed in the IBS-D. Treatment with NF-κB inhibitor pyrrolidine dithiocarbamate (PDTC) for 1 h decreased the XIST level, *versus* the dimethyl sulfoxide control (Fig. 4*B*). To explore the role of NF-κB and XIST *in vitro*, we treated the LPS-treated NCM460 cells with PDTC with or without XIST overexpression simultaneously. Western blot data (Fig. 4*C*) indicated that PDTC treatment (1 h) diminished the levels of NF-κB p65 and p-NF-κB p65 in LPS-treated NCM460 cells. The qRT-PCR analysis (Fig. 4*D*) showed XIST was downregulated in response to PDTC in LPS-treated NCM460 cells.

CCK-8 assay and flow cytometry were then performed to detect the cell viability and apoptosis in LPS-treated NCM460 and CMEC cells in response to different treatments. According to the results, PDTC treatment (1 h) alone led to enhanced viability and attenuated apoptosis in the LPS-treated cells, while additional XIST overexpression reversed the effects of PDTC (Fig. 4, *E*–*H*). Then, cytokines and tight junction proteins were determined by ELISA, qRT-PCR, and Western blot (Fig. 4, *I*–*K*). It was found that PDTC augmented the cell viability, repressed the apoptosis, downregulated the IL-1β and IL-6, and elevated the expression of ZO-1 and occludin in LPS-treated NCM460 cells; whereas, additional XIST overexpression abolished the aforementioned effects of PDTC. Further detection of TER revealed that PDTC treatment (1 h) enhanced the tight junction integrity that had been damaged by LPS, while XIST overexpression abrogated the tight junction integrity-promoting effect of PDTC treatment (1 h) alone (Fig. 4*L*).

In conclusion, inhibition of NF-κB suppresses XIST, thereby promoting enterocyte viability, inhibits apoptosis and cytokine production, and maintains tight junction integrity.

### miR-16 inhibits TLR4/NF-κB pathway, thus repressing XIST to augment enterocyte viability, inhibit apoptosis and cytokine production, and maintain tight junction integrity

**Figure 5:**
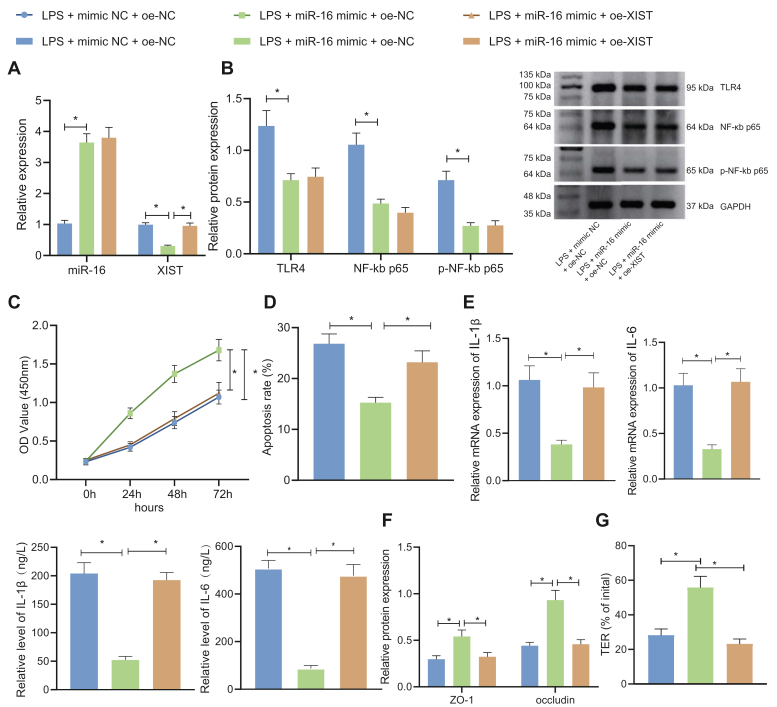
**miR-16 inhibits the TLR4/NF-κB pathway, thus repressing XIST to augment cell viability, maintain tight junction integrity, and inhibit apoptosis and cytokine production in LPS-induced NCM460 cells.***A*, qRT-PCR for determination of miR-16 and XIST level in LPS-induced NCM460 cells treated with miR-16 mimic alone or combined with oe-XIST. *B*, Western blot detection of TLR4, NF-κB p65, and p-NF-κB p65 levels in LPS-induced NCM460 cells treated with miR-16 mimic alone or combined with oe-XIST. *C*, CCK-8 assay of LPS-induced NCM460 cell viability following treatment with miR-16 mimic alone or combined with oe-XIST. *D*, flow cytometry of LPS-induced NCM460 cell apoptosis following treatment with miR-16 mimic alone or combined with oe-XIST. *E*, ELISA and qRT-PCR for detection of IL-1β and IL-6 level in the LPS-induced NCM460 cell culture medium following treatment with miR-16 mimic alone or combined with oe-XIST. *F*, Western blot detection of expression of tight junction proteins ZO-1 and occludin in LPS-induced NCM460 cells treated with miR-16 mimic alone or combined with oe-XIST. *G*, TER was determined to reflect the tight junction integrity in the LPS-induced NCM460 cells treated with miR-16 mimic alone or combined with oe-XIST. ∗*p* < 0.05. Measurement data were expressed as mean ± SD. Comparison of data among multiple groups was performed by one-way ANOVA, followed by Tukey’s post hoc test. Statistical analysis concerning time-based measurements within each group was realized using repeated measures ANOVA with Bonferroni’s post hoc test. All cell experiments were conducted independently in triplicate. CCK-8, cell-counting kit-8; LPS, lipopolysaccharide; PDTC, pyrrolidine dithiocarbamate; qRT-PCR, quantitative RT-PCR; TER, transepithelial resistance; XIST, X-inactive specific transcript.

To investigate the effects of miR-16/TLR4/XIST on the NCM460 cells, we overexpressed miR-16, or together with XIST, in the LPS-treated NCM460 cells. The qRT-PCR (Fig. 5*A*) and Western blot (Fig. 5*B*) showed that miR-16 mimic transfection diminished expression of TLR4, NF-κB p65, p-NF-κB p65, and XIST in LPS-treated NCM460 cells. Further overexpressing XIST resulted in no remarkable changes in the level of miR-16, TLR4, and NF-κB p65. CCK-8 assay and flow cytometry were conducted to unveil the cell viability and apoptosis (Fig. 5, *C* and *D*), while cytokines and tight junction proteins were examined by ELISA, qRT-PCR, and Western blot (Fig. 5, *E* and *F*). miR-16 mimic alone resulted in increased cell viability, decelerated the apoptosis, suppressed the IL-1β and IL-6, and enhanced the expression of ZO-1 and occludin, and simultaneous overexpression of XIST reversed the effects of miR-16 overexpression alone. Consistently, the tight junction integrity-promoting effect of miR-16 mimic alone, as reflected by increased TER level, was reversed when miR-16 mimic was combined with XIST restoration (Fig. 5*G*).

The aforementioned data demonstrated that miR-16 inhibited the TLR4/NF-κB pathway to repress XIST, enhanced enterocyte viability, repressed apoptosis and cytokine production, and maintained tight junction integrity.

### miR-16 inhibits TLR4/NF-κB pathway, thus repressing XIST to relieve IBS *in vivo*

**Figure 6:**
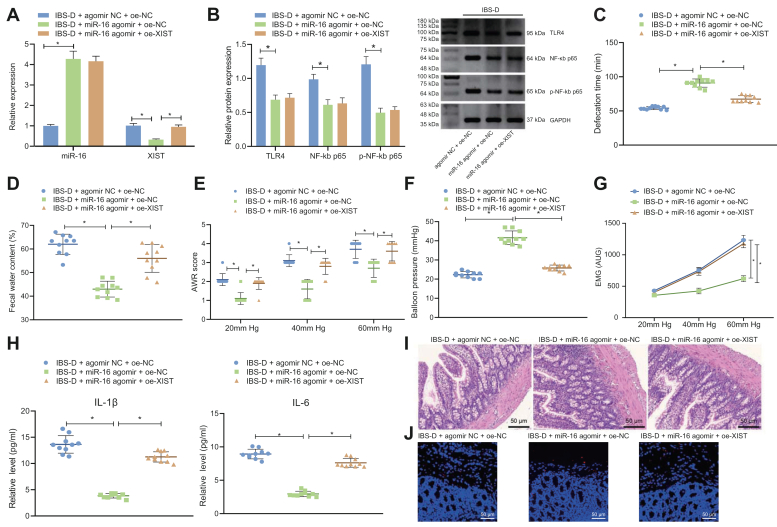
**miR-16 inhibits the TLR4/NF-κB pathway, thus repressing XIST to relieve IBS in mice.***A*, qRT-PCR for determination of miR-16 and XIST level in the colorectal tissues of IBS-D mice treated with miR-16 agomir alone or combined with oe-XIST. *B*, Western blot detection of TLR4, NF-κB p65, and p-NF-κB p65 levels in the colorectal tissues of IBS-D mice treated with miR-16 agomir alone or combined with oe-XIST. *C*, defecation intervals of IBS-D mice treated with miR-16 agomir alone or combined with oe-XIST. *D*, stool types of IBS-D mice treated with miR-16 agomir alone or combined with oe-XIST. *E*, AWR scores of IBS-D mice treated with miR-16 agomir alone or combined with oe-XIST. *F*, detection colorectal stepwise distention of IBS-D mice treated with miR-16 agomir alone or combined with oe-XIST. *G*, detection of EMG activity of IBS-D mice treated with miR-16 agomir alone or combined with oe-XIST. *H*, ELISA detection of IL-1β and IL-6 levels in the tissue homogenate of IBS-D mice treated with miR-16 agomir alone or combined with oe-XIST. *I*, H&E staining detection of pathological conditions of rectal mucosa of IBS-D mice treated with miR-16 agomir alone or combined with oe-XIST. *J*, TUNEL staining was used to detect the apoptosis in the colorectal tissues of IBS-D mice treated with miR-16 agomir alone or combined with oe-XIST. ∗*p* < 0.05. Measurement data were expressed as mean ± SD. Comparison of data among multiple groups was performed by one-way ANOVA, followed by Tukey’s post hoc test. n = 10. AWR, abdominal withdrawal reflex; IBS, irritable bowel syndrome; IBS-D, IBS with diarrhea; qRT-PCR, quantitative RT-PCR; XIST, X-inactive specific transcript.

To verify that miR-16 relieved IBS through the XIST axis, we overexpressed miR-16, or together with XIST, in the colorectal tissue of the IBS-D mice. The results of qRT-PCR and Western blot (Fig. 6, *A* and *B*) showed that overexpressing miR-16 suppressed the TLR4, NF-κB p65, and XIST in IBS-D mice. Further overexpression of XIST resulted in no notable difference in miR-16, TLR4, NF-κB p65, and p-NF-κB p65 levels but upregulated XIST expression in the presence of miR-16 overexpression.

**Supplemental Figure S1:**
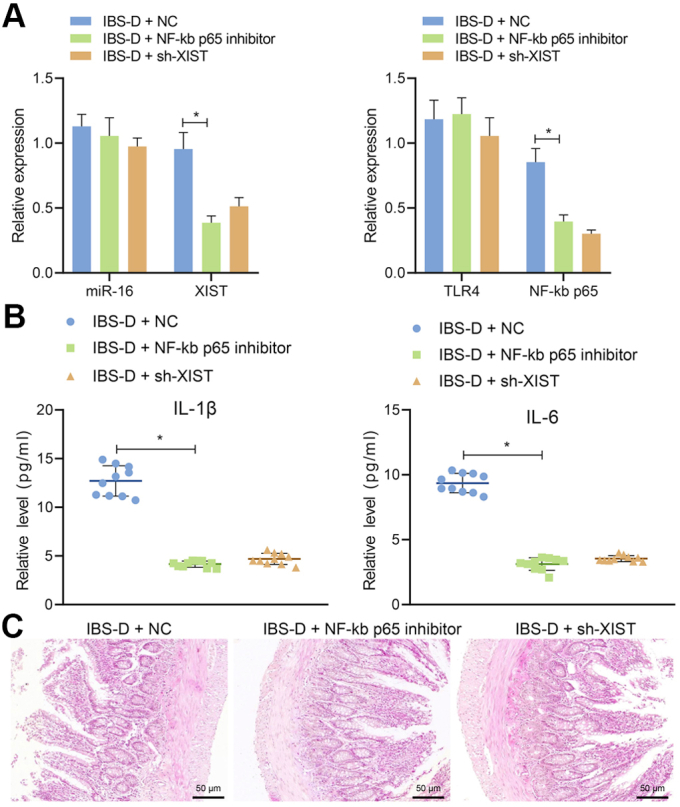
**Effects of NF-κB p65 inhibitor and sh-XIST on IBS-D mice.***A*, the miR-16 and XIST expressions as detected by RT-qPCR (*left panel*) and the expressions of TLR4 and NF-κB p65 as detected by Western blot (*right panel*) in colonic mucosal tissues of IBS-D mice in response to NF-κB-inhibitor or sh-XIST. *B*, the levels of IL-1β and IL-6 in serum of as detected by Western blot (*right panel*) as detected by ELISA; *C*, HE staining was used to observe the pathological changes of rectal mucosa of IBS-D mice in response to NF-κB-inhibitor or sh-XIST. ∗*p* < 0.05.

Next, we treated IBS-D mice with NF-κB p65 inhibitor and sh-XIST, respectively. According to the RT-qPCR and Western blot results, expression of XIST and NF-κB p65 was diminished in the NF-κB p65 inhibitor presence, and XIST was only reduced in sh-XIST presence (Fig. S1*A*). The results of ELISA showed that NF-κB p65 inhibitor inhibited the serum levels of IL-1β and IL-6 in IBS-D mice (Fig. S1*B*). In addition, H&E staining found that compared with the untreated IBS-D mice, the inflammatory cell infiltration was significantly reduced in the rectal mucosal tissues of NF-κB p65 inhibitor-treated IBS-D mice (Fig. S1*C*).

Defecation intervals, stool types, and AWR scores of the mice were evaluated and summarized in Figure 6, *C*–*E*. IBS-D mice transduced with miR-16 agomir showed extended defecation intervals, decreased water content in stool, and raised AWR scores. Overexpression of XIST reversed symptoms aforementioned. Results of colorectal stepwise distention (Fig. 6*F*), EMG activity (Fig. 6*G*), and expressions of IL-1β and IL-6 (Fig. 6*H*) indicated increased injury threshold, eliminated EMG, and suppressed IL-1β and IL-6 in the IBS-D mice transduced with miR-16 agomir. Overexpressing the XIST level aggravated the symptoms of mice. H&E staining was conducted to evaluate the histological change of the intestinal mucosa (Fig. 6*I*). No inflammatory and other pathological damage in the rectum was observed. The results of TUNEL staining then showed that the number of apoptotic cells was reduced in the presence of miR-16 agomir alone, the effects of which could then be abrogated overexpression (Fig. 6*J*).

All in all, we settled down with the conclusion that miR-16 inhibited the TLR4/NF-κB/XIST axis to relieve IBS-D.

## Discussion

**Figure 7:**
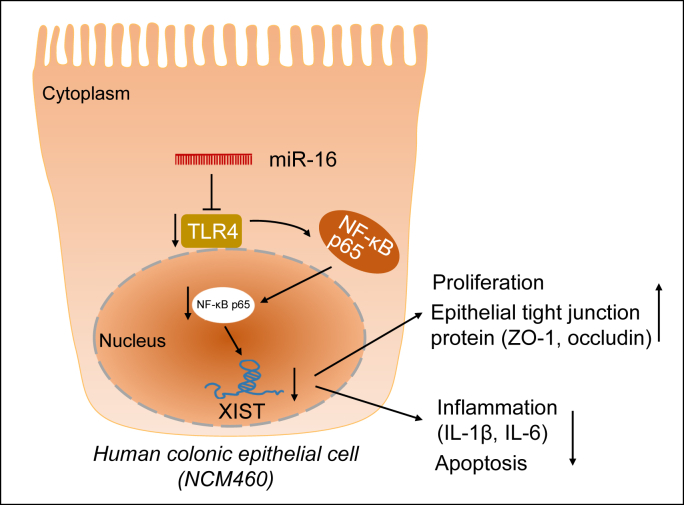
**The mechanism graph of the regulatory network of miR-16 in human colon mucosal epithelial cell (NCM460).** miR-16 targets TLR4 to suppress NF-κB p65 and the downstream XIST, resulting in accelerated cell proliferation and tight junction integrity maintenance and diminished inflammation and apoptosis. XIST, X-inactive specific transcript.

IBS is a relapsing inflammatory disorder of the gastrointestinal tract that can lead to Crohn’s disease and ulcerative colitis (16). Evidence exists reporting that underexpression of miR-16 occurs in IBS-D, and miR-16 was involved in barrier function dysregulation through the modulation of Cldn2 and cingulin expression in IBS (6). Thus, efforts of our work are focusing on deciphering the molecular mechanisms underlying effect of miR-16 in the development of IBS-D. Our findings indicated that miR-16 inhibited the TLR4/NF-κB pathway to suppress XIST, augment intestinal epithelial cell viability, inhibit the apoptosis and inflammatory cytokines, and maintain tight junction integrity in IBS-D (Fig. 7). Understanding the regulation of miR-16 holds potential to efficient prevention and therapeutic strategies for IBS-D.

Emerging evidence has pointed out the involvement of miRNAs in modulating specific biological processes in IBS-D, which may be translated clinically to restore intestinal functions and alleviate gastrointestinal symptoms (17, 18). This present study revealed that miR-16 was underexpressed in the IBS-D patients and mouse model. Corroborating finding has been identified in a previous study by Wohlfarth *et al.* (19), which demonstrated that miR-16 was poorly expressed in the jejunum of IBS-D patients. Further exploration unveiled that miR-16 overexpression enhanced enterocyte viability, restricted apoptosis and cytokine production, and maintained tight junction integrity in IBS-D. It is noted that deregulation of miR-16 expression is associated with intestinal disorders through mediating its target mRNAs (6). For a specific miRNA, the targets were various and have to be validated following *in silico* prediction. Herein, we predicted and verified TLR4 as a miR-16 target. A similar study has indicated the value of miR-16 in preventing acute lung injury through targeting TLR4 (20). Kocak *et al.* (21) have also found aberrant upregulation of TLR4 levels in IBS-D patients, which was observed to link with immune disorder along with oxidative stress.

We moved on to explain the downstream mechanisms and found that the effect of miR-16 was realized by inhibiting the TLR4/NF-κB signaling pathway. A wide array of miRNAs has been proposed to modulate intestinal inflammatory reactions through the NF-κB signaling pathway. In IBS-related ulcerative colitis, miR-126 (22), miR-150 (23), and miR-155 (24) positively regulate and induce inflammation *via* the NF-κB signaling cascade. Consistent with our findings, it is reported that miR-16 targets and inhibits TLR4 in LPS-induced inflammatory pathway but the alteration is reversed by a lncRNA (25). He *et al.* (26) corroborated that expression levels of TLR4 and NF-κB were upregulated in IBS-D rats together with increased IL-8, TNFα, and myeloid differentiation factor 88 (MyD88), but the downstream effector of NF-κB was not identified. In parallel, it is documented that TLR4 activates NF-κB to upregulate cystathionine β synthetase and increase visceral hypersensitivity in an animal model of IBS (11). In relation to our findings, a number of previous studies have indicated the inhibitory effect of miR-16 on inflammation through TLR4/NF-κB signaling (20, 21, 27). For instance, miR-16 has been recognized as mediator of inflammatory responses through downregulating the transcription level of TLR4 and interleukin-1 receptor-associated kinase 1 (IRAK-1) (28). Further, miR-16 can downregulate the expression of NF-κB, NLRP3, and other inflammatory factors by targeting TLR4, thereby attenuating the inflammation in a LPS-induced acute lung injury model (20), and sepsis mice treated with miR-146a presented with decreased NF-κB activation as well as splenocyte apoptosis (29). Although the present study intersects with previous documentation in regard of miR-16 targeting TLR4/NF-κB signaling, it stands out for expanding the relatively well-recognized mechanism to IBS, indicating the promising potential of miR-16 in this new field.

Further mechanistic investigations clarified that inhibition of NF-κB suppressed XIST to augment enterocyte viability, inhibit apoptosis and cytokine production, and maintain tight junction integrity. XIST is a 17 kb lncRNA located in the nucleus and maintains X chromosome inactivation (30). Prior evidence has suggested that silencing of XIST along the NF-κB pathway could halt LPS-induced inflammation in the lung (31). The mechanism underlying XIST expression in IBS-D has rarely been examined. We clarified that NF-κB positively related to the XIST level in IBS-D. Tight junction protein ZO-1 is deemed as an important scaffold protein, functioned as a barrier between the interior of the organism and the extracellular environment (32), and occludin was first identified in epithelial cells as an integral plasma membrane enzyme localized at the tight junction barrier. Increased level of occludin, together with ZO-1, is a sign of IBS-D mitigation. In IBS-D rats, miR-144 increases intestinal permeability and attenuates epithelial barrier function by directly targeting occludin and ZO-1 (33). It should also be noted that in this study, we used PDTC to inhibit NF-κB signaling pathway. Although PDTC is commonly applied as a NF-κB inhibitor (34, 35), it is not a NF-κB-specific inhibitor and has been reported to exert diversiform roles in a variety of cell bioactivities (36, 37, 38). Herein, whether there exist other PDTC-mediated molecular mechanisms contributing to enterocyte viability may need further investigations.

Of note, our data demonstrated that overexpression of miR-16 elevated the level of occludin and ZO-1, a sign of improved tight junction in epithelial cells, the mechanism underlying which has not been well established but may be clarified from three aspects. First, another miRNA, miR-122a, has been suggested to modulate occludin degradation through tumor necrosis factor-α (TNF-α), thereby affecting the permeability of intestinal cells (39), and miR-16 has also been correlated with TNF-α (40). Therefore, miR-16 may regulate occludin and ZO-1 based on its interaction with TNF-α. On the other hand, miR-25-3p shuttled by exosomes in colorectal cancer can be transferred to vascular endothelial cells to mediate the expression of ZO-1 and occludin by targeting Kruppel-like factors (KLFs) (41), and miR-16 also serves as a regulator of KLF4 (42), so it may be speculated that miR-16 affects ZO-1 and occludin in a KLF4-dependent manner. Moreover, PKC isoforms have been implicated in the assembly of tight junctions for being able to regulate occludin and ZO-1 (43), and miR-16 has been related to PKCα, a PKC isoform (44). Hence, it is also possible that miR-16 regulates occludin and ZO through PKCα. Moreover, it has been previously documented that IL-1β precursor could be cleaved by LPS-induced protease and then be released outside the cell in the form of IL-1β (45). More recently, a report indicated that, in response to LPS induction, the release of functional IL-1β in microparticles was under a two-step regulation by GSDM-D and P2X7 (46), and it has further been revealed that IL-1β could be detected by ELISA assay in NCM460 cells exposed to LPS stimulation (47). The aforementioned evidence supports that IL-1β can be secreted out of the cells as a secreted protein under LPS induction. Ultimately, our *in vitro* and *in vivo* experiments have validated that miR-16 inhibited the TLR4/NF-κB pathway, thus repressing XIST to maintain intestinal epithelial tight junction integrity and to relieve IBS.

Collectively, the evidence acquired in the present study showed that miR-16 targeted TLR4 to suppress NF-κB and the downstream XIST, resulting in accelerated enterocyte viability, tight junction integrity maintenance, and diminished inflammation and apoptosis. We believe that miR-16 may be a promising target for preventing or treating IBS-D as well as other intestinal diseases caused by manifold dysfunction. However, despite the involvement miR-16/TLR4/NF-κB axis in IBS-D, we have not elucidated the exact location of the miRNA. miR-16 may locate in the cytoplasm, extracellular vesicles, or extracellular matrix. Moreover, the current results do not imply that miR-16 is the only miRNA involved in the NF-κB pathway against IBS-D. Other miRNAs may target XIST, ZO-1, or other effectors in the pathway, which should be studied in future investigations. Future exploration using two signals to activate inflammation, including LPS and TNFa, may help further consolidating the model and understanding the mechanistic actions. Moreover, ongoing and future studies are required to expand validation of the effect of miR-16 on invasion of inflammatory cells in tissues in IBD animal models. Notwithstanding its limitations, this study does suggest the significance of miR-16 in IBS-D.

## Experimental procedures

### Ethics statement

All the patients and healthy control participants signed informed consent documentation. The study was approved by the Ethics Committee of Changshu Hospital Affiliated to Nanjing University of Chinese Medicine and complied with the Declaration of Helsinki. Animal experiments were conducted with approval of the Animal Ethics Committee of Changshu Hospital Affiliated to Nanjing University of Chinese Medicine and in accordance with the Guide for the Care and Use of Laboratory animals published by the US National Institutes of Health.

### Bioinformatics analysis

The downstream genes of miR-16 were predicted by RNAInter. The genes were screened by the evaluation scores. Gene interactions were analyzed by STRING and visualized using Cytoscape 3.5.1. The core gene was chosen for further evaluation. IBS-related genes were searched in the GeneCards database using the keyword “irritable bowel syndrome” and plotted with the target core genes using jvenn. To further investigate the gene modulation pathway, we utilized KOBAS 3.0 for the KEGG pathway enrichment analysis. microRNA.org was adopted for analysis of the binding site for miR-16 and target genes.

### Sample collection

Patients who underwent colonoscopy examination from Changshu Hospital Affiliated to Nanjing University of Chinese Medicine from March 2016 to March 2017 were enrolled in this study, including 37 IBS-D patients and 37 healthy controls. The demographics of IBS-D patients and healthy controls are shown in Table S1. IBS-D patients were included according to the ROME III standard: (1) recurrent abdominal pain or abdominal discomfort, for at least 3 days per month for the last 3 months, accompanied by the following: bowel pain or discomfort relief after defecation, changes in defecation frequency, changes in stools characterization; (2) abdominal pain or discomfort at least twice per week. (3) IBS-D scores according to the Bristol stool chart, defecating fluffy and mushy stools for more than 25% of the period while hard and lumpy stools for less than 25% of the period. Normal subjects who were negative for the colonoscopy and patients coexisted other diseases were excluded. Peripheral blood samples of the patients were collected.

### Extraction serum total RNA

A kit (Norgen Biotek Corp, NGB-55500) was used for this experiment. Briefly, 250 μl of serum samples were mixed with 750 μl of TRIzol LS reagent and left to stand for 5 min. The mixture was then added with 0.2 ml of chloroform, shaken vigorously for 15 s, and allowed to stand at room temperature (RT) for 2 min. Next, the mixture was centrifuged at 13,000 rpm for 5 min and the supernatant was harvested, 0.5 ml of which was pipetted into a new 1.5 ml EP tube and then mixed with 0.3 ml isopropyl alcohol, put into the adsorption column, centrifuged at 13,000 rpm for 15 s. Thereafter, the column was added with 500 μl washing buffer and centrifuged at 13,000 rpm for 15 s, which was repeated again. The adsorption column was put back into the centrifuge and centrifuged at 13,000 rpm for 2 min, with the residual ethanol thoroughly shaken off. The adsorption cartridge was placed into a 1.5 ml nuclease-free collection tube, where 20 to 50 μl nuclease-free H_2_O was supplemented and allowed to stand at RT for 2 min, followed by centrifugation at 13,000 rpm for 1 min. The eluate was the RNA product and cryopreserved. Finally, the RNA concentration was determined using a NanoDrop spectrophotometer.

### qRT-PCR

Total RNA was extracted using TRIzol reagent and reversely transcribed into complementary DNA (cDNA) using TaqMan MicroRNA Assays Reverse Transcription primer (4427975, Applied Biosystems) and PrimeScript RT kit (Takara). Meanwhile, a PolyA tailing kit (B532451, Sangon Biotech) was utilized to generate cDNA from miRNA. qRT-PCR was performed with Fast SYBR Green PCR kit (Applied Biosystems) and ABI 7500 RT-PCR system (Applied Biosystems). U6 and GAPDH served as internal standards. The 2^−ΔΔCT^ method was used to calculate the relative expression of genes. Primer sequences are listed in Table S2.

### Establishment of IBS-D mouse model

NIH mice at a specific pathogen-free level, with both genders equally divided, were purchased from the Experimental Animal Center of Changshu Hospital Affiliated to Nanjing University of Chinese Medicine. The mice were randomized into four experimental groups and one control group, with ten mice in each group. The IBS-D mouse model was established by acetic acid instillation (48, 49). After fed for 7 days, the control mice were instilled with distilled water, while the model mice were instilled with acetic acid (0.5 ml per day). The model was evaluated after instillation for 14 days. The stool type and gastrointestinal transit time of each group were recorded. Colonic sensitivity test and AWR scores were used for visceral sensitivity assessment. Histologic changes in colon mucosa were detected using H&E staining. Animal experiments were conducted following the protocols approved by the Animal Care and Use Committee of Changshu Hospital Affiliated to Nanjing University of Chinese Medicine and following the National Institutes of Health guidelines.

The mouse model was evaluated (50) as follows: (1) defecation time: each mouse was given 0.5 ml phenol red by intragastric administration. The time from gavage to red feces defecation was recorded. (2) Fecal water content: each mouse was given 0.5 ml phenol red by intragastric administration. Feces were collected for 2 h after gavage, and the wet weight was measured. The corresponding dry weight was measured after air dried. Fecal water content was calculated, that is, fecal water content = (wet weight − dry weight)/wet weight of feces × 100%. (3) Visceral sensitivity: the colorectal distension was performed by placing a balloon in the rectum. The airbag was placed 2 cm from the anus. After adaptation, the air was quickly injected into the airbag to a specified pressure (20, 40, or 60 mmHg) and maintained 20 s. Experiments were repeated five times at each pressure with an interval of 4 min. Evaluators, without the knowledge of grouping and air pressure, independently and synchronously observed the mice AWR and recorded the scores. Averaged AWR was calculated to indicate visceral sensitivity. AWR scales are as follows: AWR0: no remarkable behavior response; AWR1: occasionally head movement with an immobilized body; AWR2: mild abdominal muscle contraction without uplift; AWR3: abdominal muscle contraction with uplift; AWR4: pelvis uplift with an arched spine.

The IBS-D mice were grouped into (1) IBS-D + agomir NC + oe-NC group (injected with adenovirus vector containing agomir negative control and adenovirus vector containing overexpression negative control), (2) IBS-D + miR-16 agomir + oe-NC group (injected with adenovirus vector containing miR-16 agomir and adenovirus vector containing overexpression negative control), and (3) IBS-D + miR-16 agomir + oe-XIST group (injected with adenovirus vector containing miR-16 agomir and adenovirus vector containing XIST overexpression). Adenovirus vectors with the titer of 2 × 10^8^ PFU/ml were prepared and inoculated under the left axilla of the mice using a 1 ml syringe. Afterward, the mice were housed in a specific pathogen-free laboratory for the subsequent experiments.

### Injurious visceral hypersensitivity

The balloon was fixed on the pipe connected to an automatic expansion device (G&J Electronic) for colon dilatation. After lubrication, the balloon was placed into the distal colon of the mice with the top of the balloon 0.5 to 1 cm from the anus. The mice were confined to plastic containers and allowed to adaption for 15 to 20 min before testing. Stepwise distention in the colon was given from 0 to 60 mmHg with a step of 5 mmHg until the first contraction of the testicles, tail, or abdominal muscles, which was defined as the injurious visceral hypersensitivity pain threshold. Colon dilatation was repeated within 5 to 10 min. Stimulus intervals and the averaged pressure for each mouse were recorded.

Lateral oblique EMG was examined to determine the visceral sensitivity of the IBS-D mice. The mouse was anesthetized with an i.p. injection of 50 mg/kg pentobarbital sodium. Two electrodes were implanted in the lateral oblique and externalized at the back of the head. The colorectal was dilatated to 20, 40, or 60 mmHg for 20 s and repeated after a 2 min interval. EMG was recorded on an electromyogram amplifier module EMG 100C (Biopac Systems).

### ELISA

The cell supernatant or tissue homogenizer was collected. ELISA kits were applied for quantification of IL-1β and IL-6 (BMS224-2 and BMS213-2, Thermo Fisher). Absorbance at 450 nm was recorded using a microplate reader (Wallac 1420 Multilabel, PerkinElmer).

### H&E staining

Mouse rectal tissue was embedded with paraffin wax, sliced, treated with xylene I for 10 min, and stirred in xylene II for 10 min. The section was then rinsed in absolute ethanol/xylene (v/v 1/1) for 1 min, dewaxed and hydrated, stained with hematoxylin for 10 min, and differentiated in 0.25% hydrochloric acid alcohol for 3 s. Afterward, the slides were washed in ethanol for 1 min, stained with 0.5% eosin solution for 1 min, washed with 3% ethanol for 30 s before gradient alcohol dehydration, and being rendered transparent by absolute ethanol/xylene (1:1) for 1 min. The sections were rinsed in the xylene I and II for 5 min each, sealed with neutral gum in the ventilation cabinet, and observed under an optical microscope (Olympus).

### TUNEL staining

Prepared slides of the colorectal tissue were treated with 0.1% Triton X-100 in PBS, subjected to 2 min ice bath, permeabilized, and then treated with 50 μl TUNEL detection solution, followed by 60 min incubation (37 °C) in the dark. After mounting with antifluorescence quenching solution, the slides were observed with a fluorescence microscope (FV1000, Olympus), with an excitation wavelength ranging from 450 to 500 nm and an emission wavelength from 515 to 565 nm. Cells presenting with red fluorescence were recognized as apoptotic cells.

### Cell culture

Human normal colonic epithelial cell line NCM460 was purchased from Biobw (bio-108818) and cultured in RPMI 1640 medium supplemented with 10% fetal bovine serum (FBS) and 1% penicillin and streptomycin (Sigma–Aldrich) in the incubator (37 °C, 5% CO_2_). A human colonic mucosal epithelial cell line CMEC was purchased from Biobw (bio-73503) and cultured in Dulbecco's modified Eagle's medium (DMEM) supplemented with 10% FBS. Human embryonic kidney cells HEK-293T (CRL-11268, ATCC) were cultured in DMEM under the same condition. Cells in the logarithmic growth period were digested with trypsin and seeded in a 6-well plate with 1 × 10^5^ cells per well. Cells were cultured in the complete medium for 24 h until the confluence reached about 75%. Transfection was performed using Lipofectamine 2000. After 48 h of transfection, the efficiency was examined by qRT-PCR. Cells were grouped into a mimic NC group, a miR-16 mimic group, an oe-NC group, an oe-TLR42 group, and an oe-XIST group. Plasmids for silencing were synthesized from GenePharma (pGPU6/Neo, C02003). Plasmids for overexpression (pCDNA3.1- FLAG-LPA2 in overexpression groups) were purchased from Miaolingbio (P1224) and those for miR-16 inhibitor and its corresponding NCs were from GenePharma. We established the LPS-induced IBS-D model in the normal colonic epithelial cell line NCM460 (15). LPS concentration was 2 μg/ml, which was used as the control for LPS induction. The final concentration of dimethyl sulfoxide (as control) and TLR4 inhibitor TAK-242 (Merck Millipore) was 1 μM and that of NF-κB inhibitor PDTC was 10 μM. The treatment time was 1 h.

### CCK-8 assay of cell viability

Cells were digested and suspended after 48 h of transfection. The cell density was adjusted to 1 × 10^5^ cells/ml, and 100 μl of cell suspension was seeded into 96-well plates. The cells were cultured overnight. The cell viability was tested at 0, 24, 48, and 72 h after inoculation using CCK-8 kit (Beyotime). For the measurement, 10 μl CCK-8 reagent was added to each well and the cells were incubated for 4 h before the absorbance at 450 nm was recorded. The cell growth curve was meanwhile plotted.

### Flow cytometric detection of cell apoptosis

Cells were collected by centrifugation at 2000*g* for 5 min. The medium was discarded. The cells were washed with cold PBS twice, suspended in the binding buffer (1×, 400 μl) with AnnexinV-FITC (5 μl), and incubated at 4 °C for 15 min in dark. The mixture was further incubated at 4 °C for 5 min in the dark after propidium iodide (10 μl) was added. The specimens were assessed in flow cytometry (FACSCalibur, BD Bioscience) within 1 h. All experiments were conducted independently in triplicate.

### Western blot

The cells were digested by trypsin, lysed with enhanced radioimmunoprecipitation assay lysis buffer containing trypsin (Boster Biotech), and quantified using bicinchoninic acid kit (Boster Biotech). The proteins were separated by SDS-PAGE and transferred onto polyvinylidene fluoride membrane. The membrane was blocked in 5% bovine serum albumin for 2 h and then incubated at 4 °C overnight with diluted primary antibodies: rabbit anti-ZO-1 (ab216880, 1:1000, Abcam), anti-occludin (ab167161, 1:5000, Abcam), anti-TLR4 (ab13556, 1:500, Abcam), anti-NF-κB p65 (ab16502, 1:2000, Abcam), anti-NF-κB p65 (phospho S536, ab76302, 1:1000, Abcam), and anti-GAPDH (ab181602, 1:5000, Abcam). After incubation, horseradish peroxidase (or AF488) labeled anti-rabbit IgG (ab6721, or ab150117, 1:2000, Abcam) was added for another 1 h incubation at RT. The immunoblots were visualized with enhanced chemiluminescence reagents (EMD Millipore). The images were captured and analyzed by ImageJ 1.48u (Bio-Rad). GAPDH was served as the internal reference. All experiments were conducted independently in triplicate.

### Dual-luciferase reporter assay

The binding sites of miR-16 and TLR4 were predicted by starBase. HEK293T cells were cultured in DMEM containing 10% FBS at 37 °C, 5% CO_2_. The TLR4 3′-UTR cDNA containing miR-16 binding site (TLR4-Wt) was inserted into the pmirGLO vector. The TLR4 3′-UTR containing mutant binding site (TLR4-Mut) was synthesis by point mutation and inserted into the pmirGLO vector. The vectors were verified by sequencing, performed by Ribobio. The two pmirGLO reconstruction vectors were transfected into HEK293T cells using liposomes together with miR-16 mimic or NC mimic. The cells were incubated for 48 h before collection and lysis. The suspension (100 μl) was mixed with Renilla luciferase detection reagent (100 μl) to check the activity of Renilla luciferase. Similarly, the Firefly luciferase activity was tested on a SpectraMax M5 (Molecular Device) for 10 s with an interval of 2 s.

### Short-circuit current detection of TER

The TER of monolayer cells were measured with the STX2 double-rod EVOM voltage resistance meter. When the NCM460 cells formed a monolayer epithelial cell barrier and the measured TER value reached a stable level, the TER of each monolayer was determined with three replicate wells for each experimental group. The measured TER level was expressed as a percentage of the initial measured level.

### Statistical analysis

Statistical data were processed by SPSS 21.0 (IBM Corp). Measurement data were presented as mean ± SD. Data comparison between two groups was performed by unpaired *t* test. Comparison of data among multiple groups was performed by one-way ANOVA with Tukey’s post hoc test. Statistical analysis concerning time-based measurements within each group was realized using repeated measures ANOVA with Bonferroni’s post hoc test. A value of *p* < 0.05 indicated a significant difference.

## Data availability

The original contributions presented in the study are included in the article/supplementary material; further inquiries can be directed to the corresponding authors.

## Supporting information

This article contains supporting information.

## Conflict of interest

The authors declare that they have no conflicts of interest with the contents of this article.

## Supporting information

Supplemental Tables S1 and S2

Original WB Images
