# Supplementary material for: MicroRNA-16 inhibits the TLR4/NF-κB pathway and maintains tight junction integrity in irritable bowel syndrome with diarrhea
Source: J Biol Chem. 2022 Sep 5;298(11):102461. doi: 10.1016/j.jbc.2022.102461 (PMC9647533; doi:10.1016/j.jbc.2022.102461)
Supplement: Supplemental Tables S1 and S2 [file mmc1.doc]

**Supplementary Table 1 Demographics of IBS-D patients and healthy controls.**

| Characteristics | Normal | IBS-D | *p* value |
| --- | --- | --- | --- |
| Average age (years) | 42.89 ±5.75 | 44.43 ±7.84 | *p* = 0.3385 |
| Sex-no. (%) |  |  | *p* = 0.4478 |
| Female | 20 (54.05%) | 24 (64.86%) |  |
| Male | 17 (45.95%) | 13 (35.14%) |  |
| Average BMI (kg/m2) | 25.15 ±0.68 | 27.72 ±0.75 | *p < 0.0001* |
| BMI-no. (%) |  |  | *p = 0.0001* |
| Underweight (≤18.5 kg/m^2^) | 0 (0.00%) | 2 (5.41%) |  |
| Healthy weight (18.6-24.9 kg/m^2^) | 16 (43.24%) | 1 (2.70%) |  |
| Overweight (25-29.9 kg/m^2^) | 19 (51.35%) | 25 (67.57%) |  |
| Obese (≥30 kg/m^2^) | 2 (5.41%) | 9 (24.32%) |  |
| Abdominal pain score | - | 5.05 ±0.94 | - |
| Bloating score | - | 4.35 ±1.14 | - |
| Bristol Stool Form | - | 6.19 ±0.66 | - |
| Stool frequency | - | 3.76 ±0.80 | - |
| Urgency score | - | 4.62± 1.14 | - |

**Note:** IBS-D, irritable bowel syndrome with diarrhea; BMI, body mass index.

**Supplementary Table 2 Primer sequences for qRT-PCR**

| Gene | Primer sequence (5’-3’) | |
| --- | --- | --- |
| (human) miR-16 | Forward | GGTGCCTTAGCAGCACGTA |
|  | Reverse | Universal primer |
| (mouse) miR-16 | Forward | GTGCCTTAGCAGCACGTAAA |
|  | Reverse | Universal primer |
| (human) U6 | Forward | CTCGCTTCGGCAGCACA |
|  | Reverse | Universal primer |
| (mouse) U6 | Forward | CTCGCTTCGGCAGCACA |
|  | Reverse | Universal primer |
| (human) XIST | Forward | ACGATCCCTAGGTGGAGATG |
|  | Reverse | CTCTCTGCACTGCTTGTAGG |
| (human) GAPDH | Forward | GAGAAGGCTGGGGCTCATTT |
|  | Reverse | AGTGATGGCATGGACTGTGG |
| (mouse) XIST | Forward | GTCCTCGCTACTCTGAACAA |
|  | Reverse | CCACTATTGCAGCAGCTTTT |
| (mouse) GAPDH | Forward | TGCACCACCAACTGCTTAGC |
|  | Reverse | GGCATGGACTGTGGTCATGAG |

**Note:** qRT-PCR, quantitative reverse transcription-polymerase chain reaction; miR-16, microRNA-16; XIST, X-inactive specific transcript; GAPDH, glyceraldehyde-3-phosphate dehydrogenase
