# Supplementary material for: MicroRNA-16 inhibits the TLR4/NF-κB pathway and maintains tight junction integrity in irritable bowel syndrome with diarrhea
Source: J Biol Chem. 2022 Sep 5;298(11):102461. doi: 10.1016/j.jbc.2022.102461 (PMC9647533; doi:10.1016/j.jbc.2022.102461)
Supplement: Original WB Images [file mmc2.docx]

**MicroRNA-16 inhibits the TLR4/NF-κB pathway and maintains tight junction integrity in irritable bowel syndrome with diarrhea**

**Running title:** miR-16/TLR4/NF-κB/XIST in IBS-D

**Meijuan Xi ^1, #^, Ping Zhao ^2, #^, Fang Li ^1, #^, Han Bao ^1^, Sijie Ding ^1^, Lijiang Ji ^2, *^, Jing Yan ^3, *^**

**^1^** Digestive System Department, Changshu Hospital Affiliated to Nanjing University of Chinese Medicine, Changshu 215500, P.R. China

**^2^** Department of Anorectal Surgery, Changshu Hospital Affiliated to Nanjing University of Chinese Medicine, Changshu 215500, P.R. China

**^3^** First Clinical Medical College, Nanjing University of Chinese Medicine, Nanjing 210036, P.R. China

**^#^** These authors contributed equally to this work.

**^*^** **Correspondence to: Lijiang Ji**, Department of Anorectal Surgery, Changshu Hospital Affiliated to Nanjing University of Chinese Medicine, Changshu 215500, No. 6, Huanghe Road, Jiangsu Province, P.R. China; **Jing Yan**, First Clinical Medical College, Nanjing University of Chinese Medicine, Nanjing 210036, Jiangsu Province, P.R. China

**E-mail:** jobnjjsyeggomqb@163.com (Lijiang Ji); yanjingyy88@163.com (Jing Yan)

**Tel.:** +86-13962340746/+86-15005187002

**Figure legends**

The original whole blot images for figures 2E, 3F, 4C, 4K, 5B and 6B.


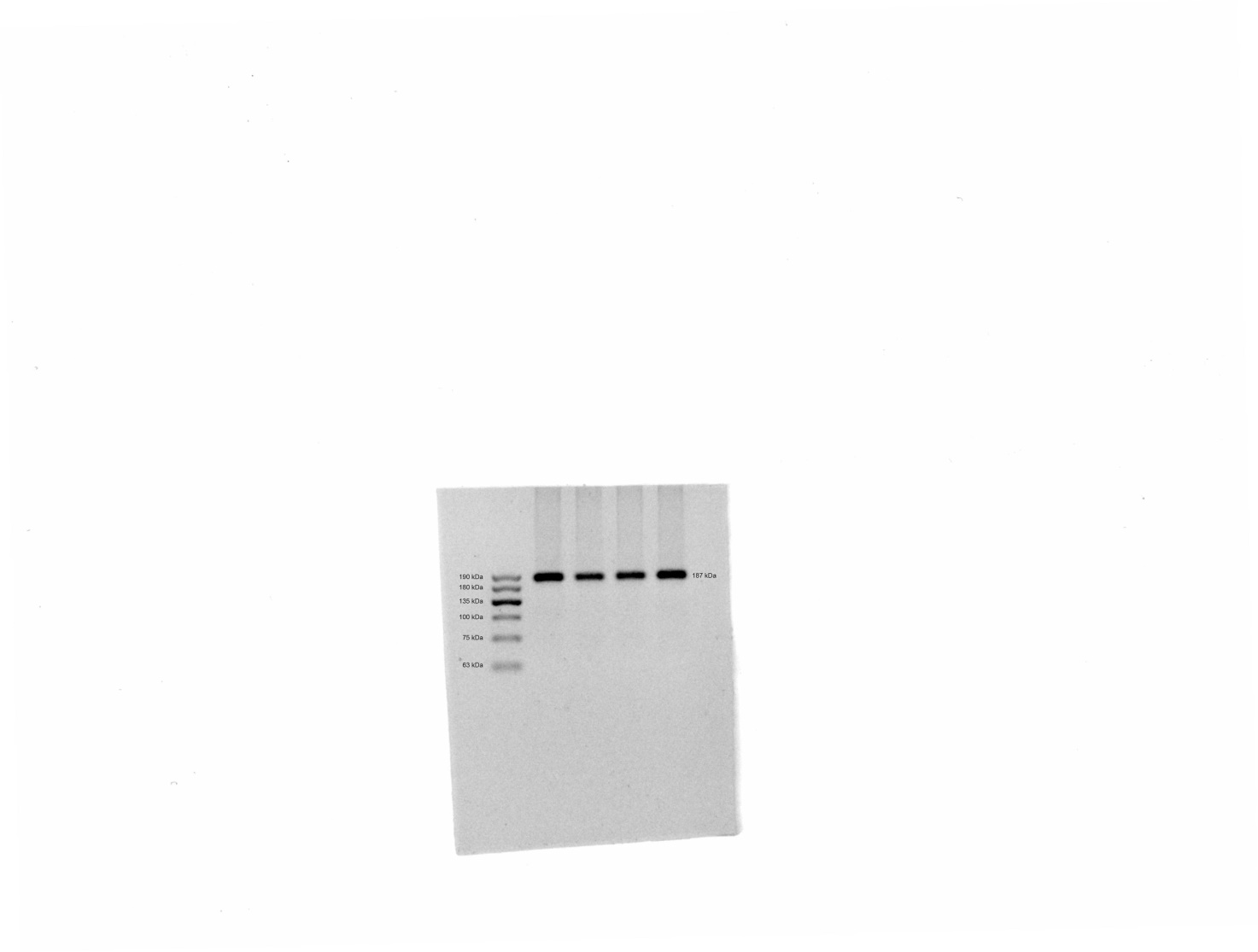


Figure2E ZO-1


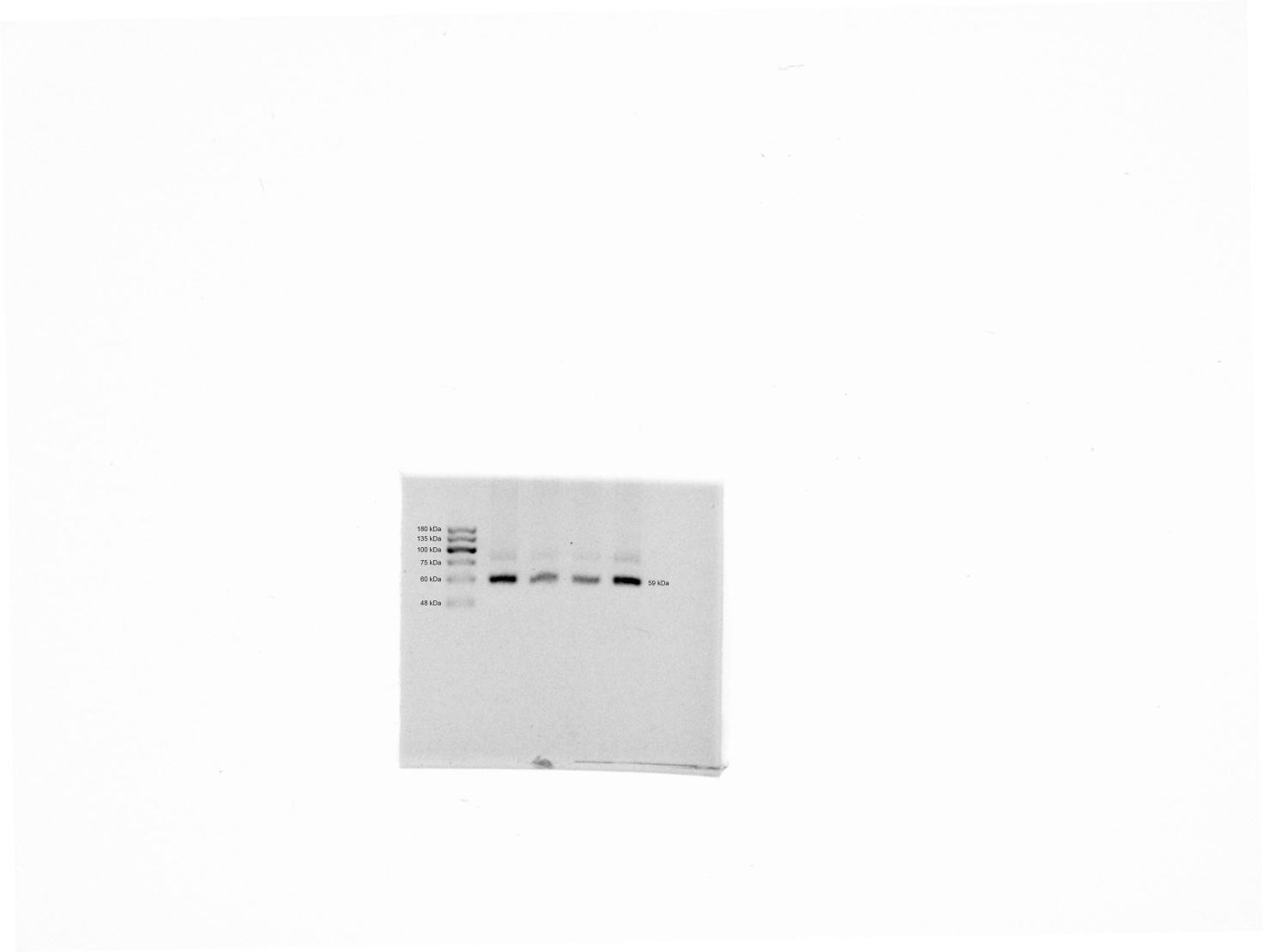


Figure2E occludin


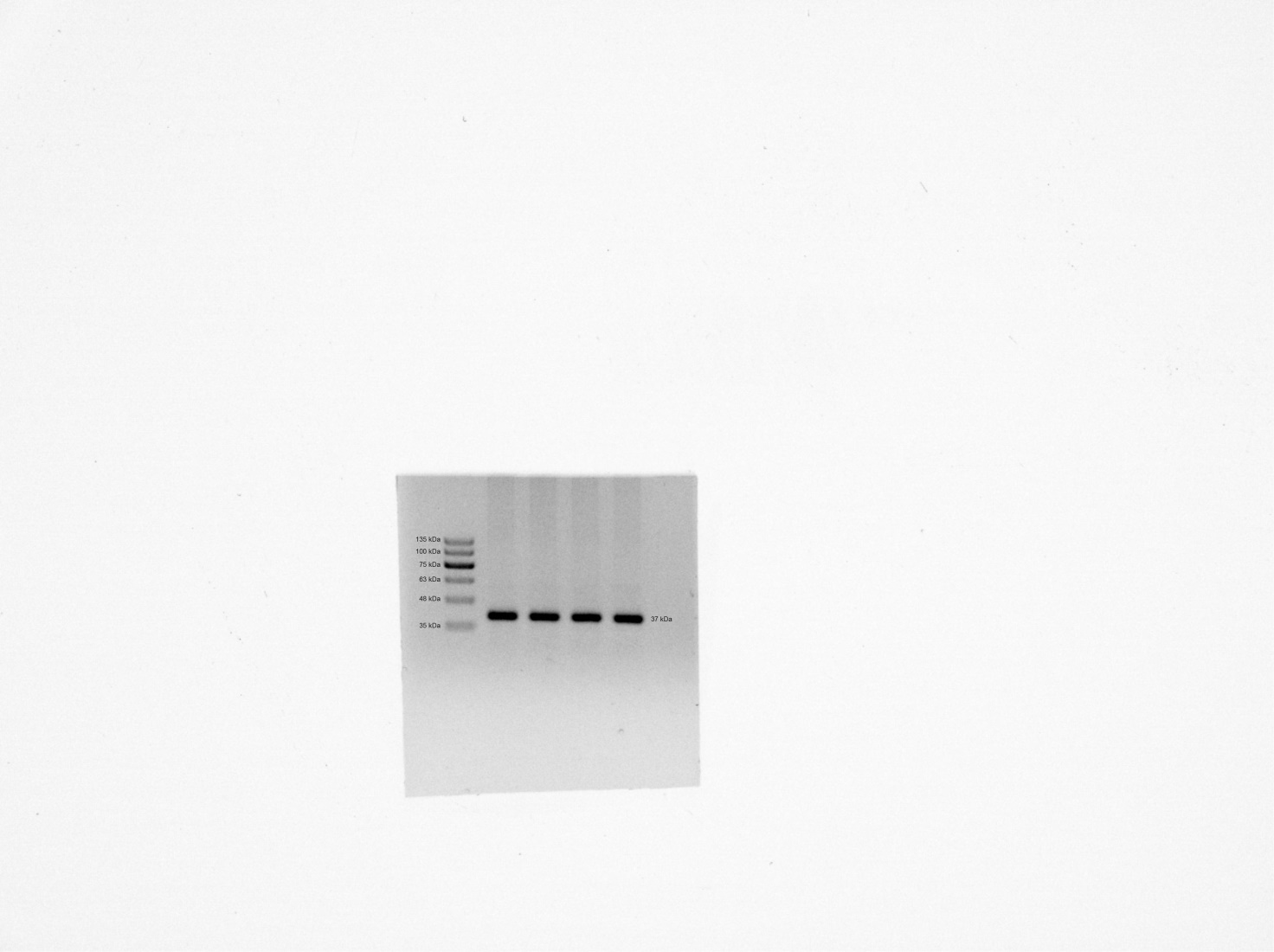


Figure2E GAPDH


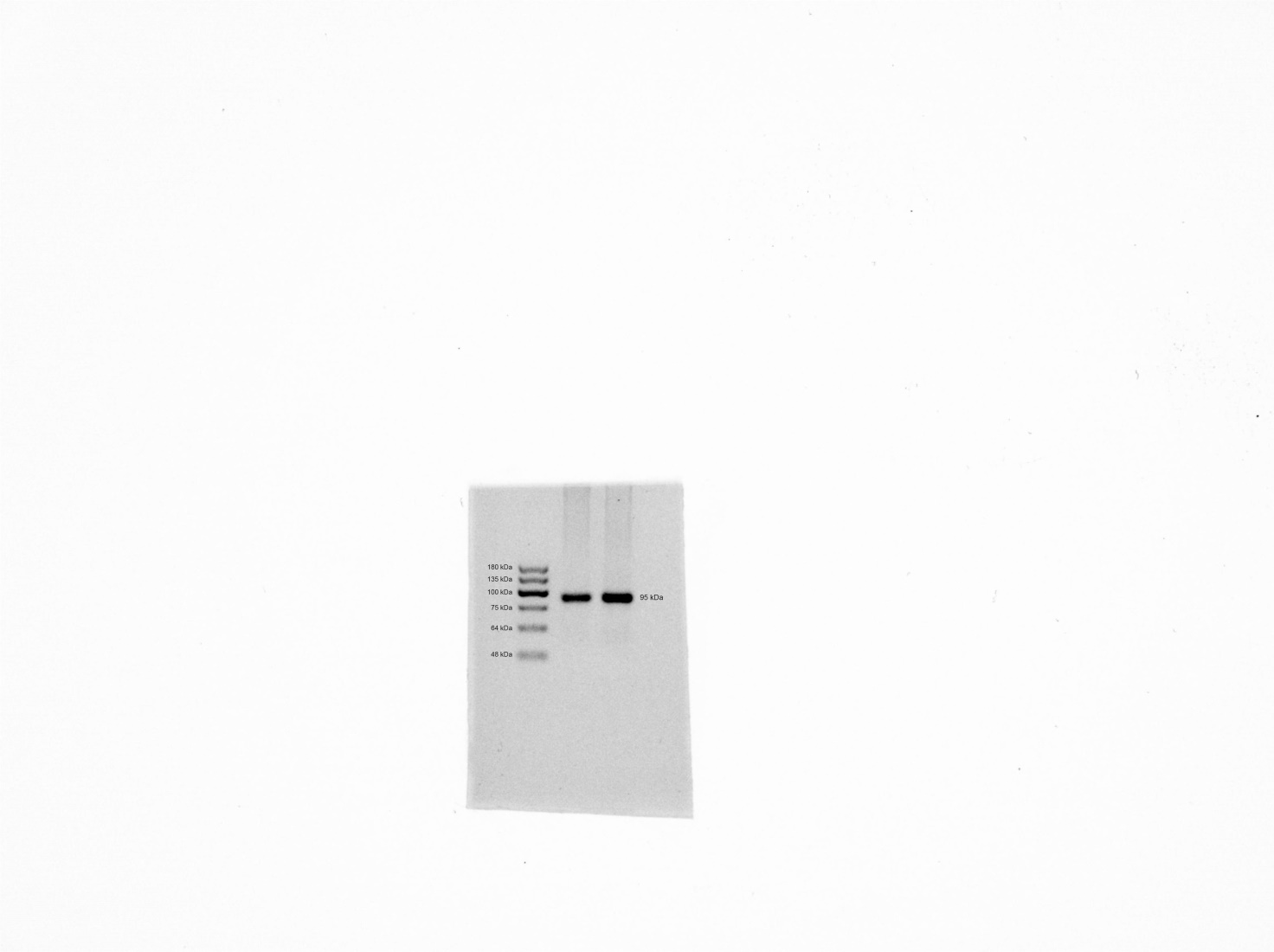


Figure3F TLR4


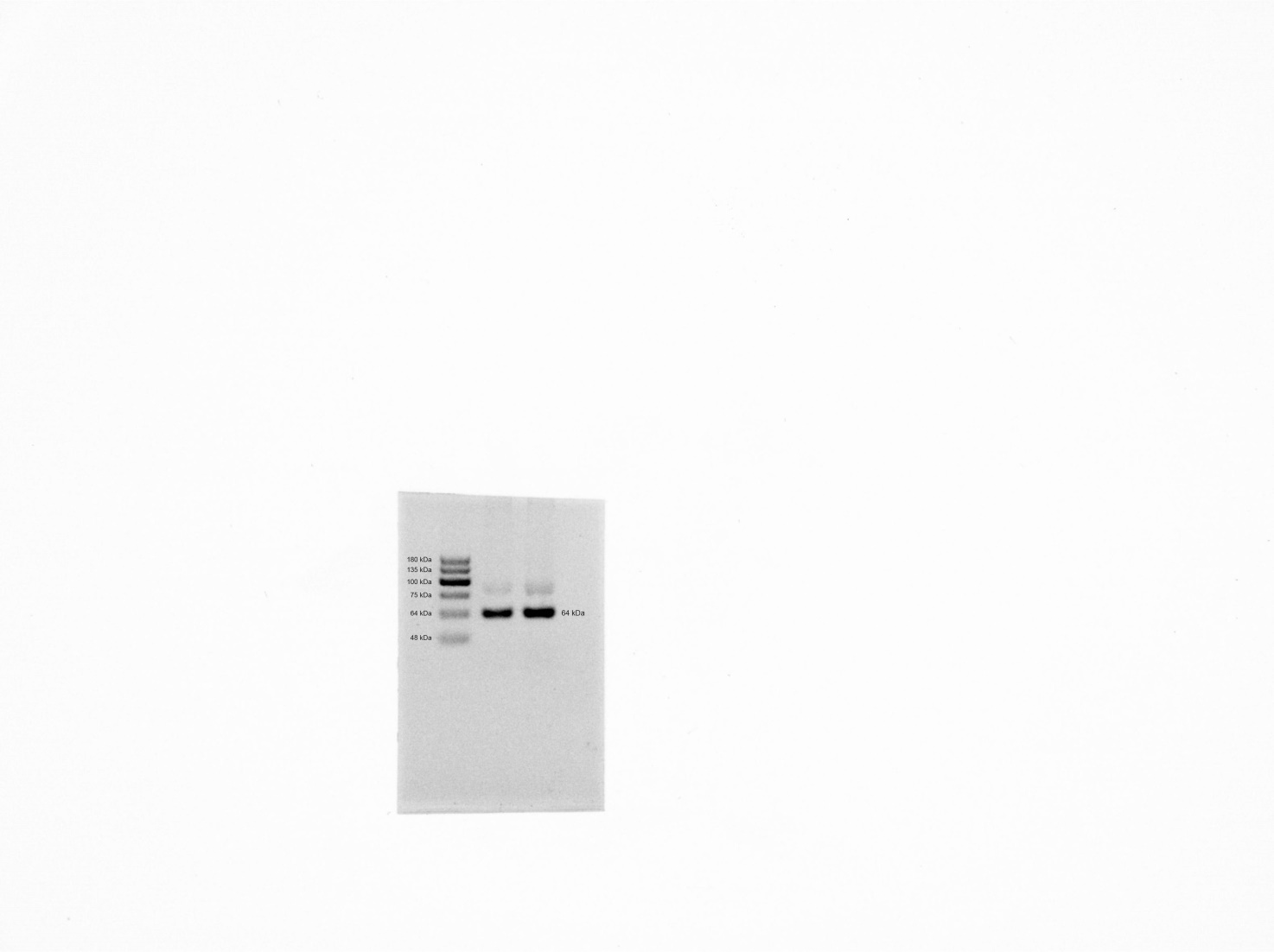


Figure3F NF-_K_B P65


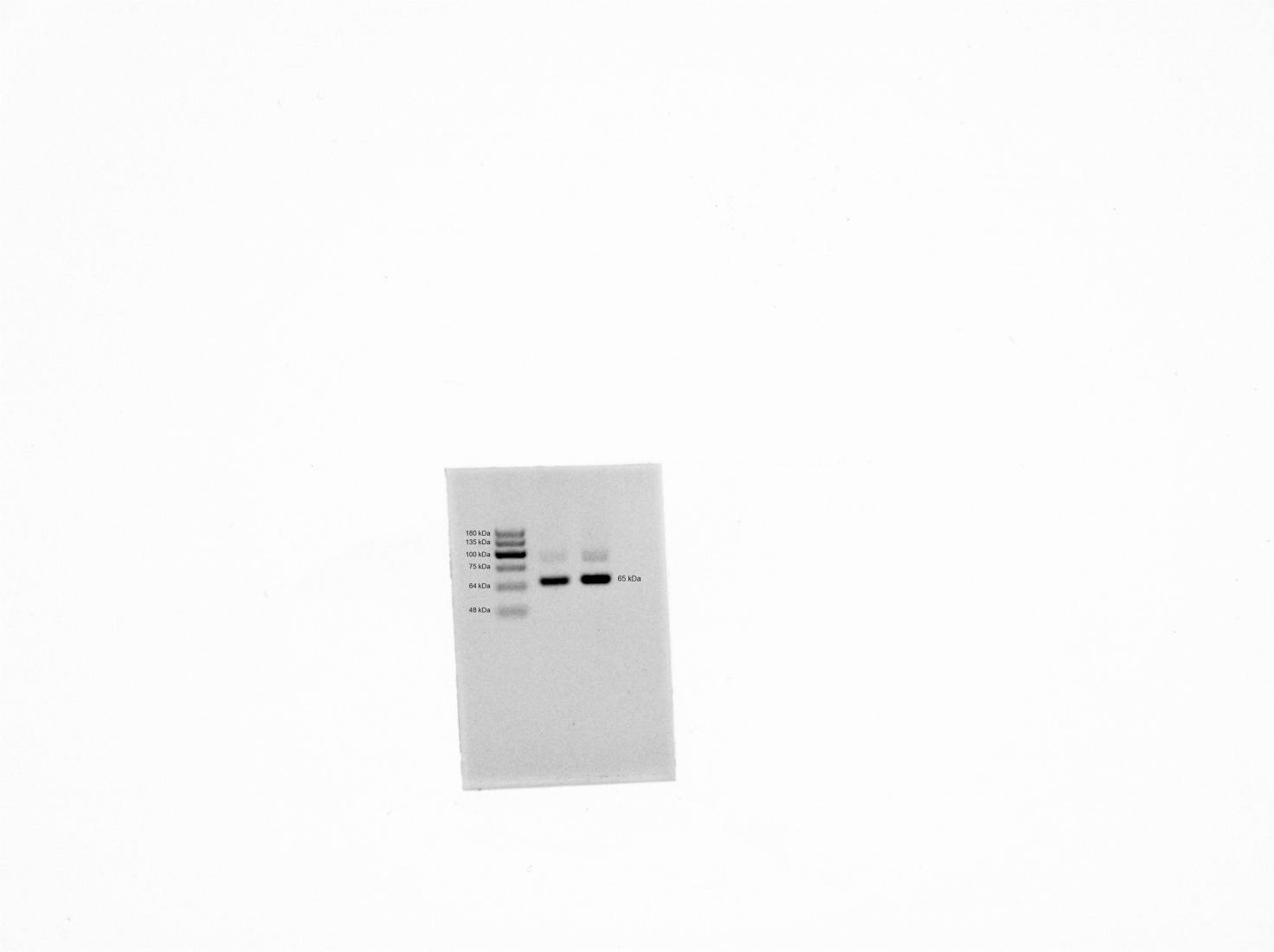


Figure3F p-NF-kb p65


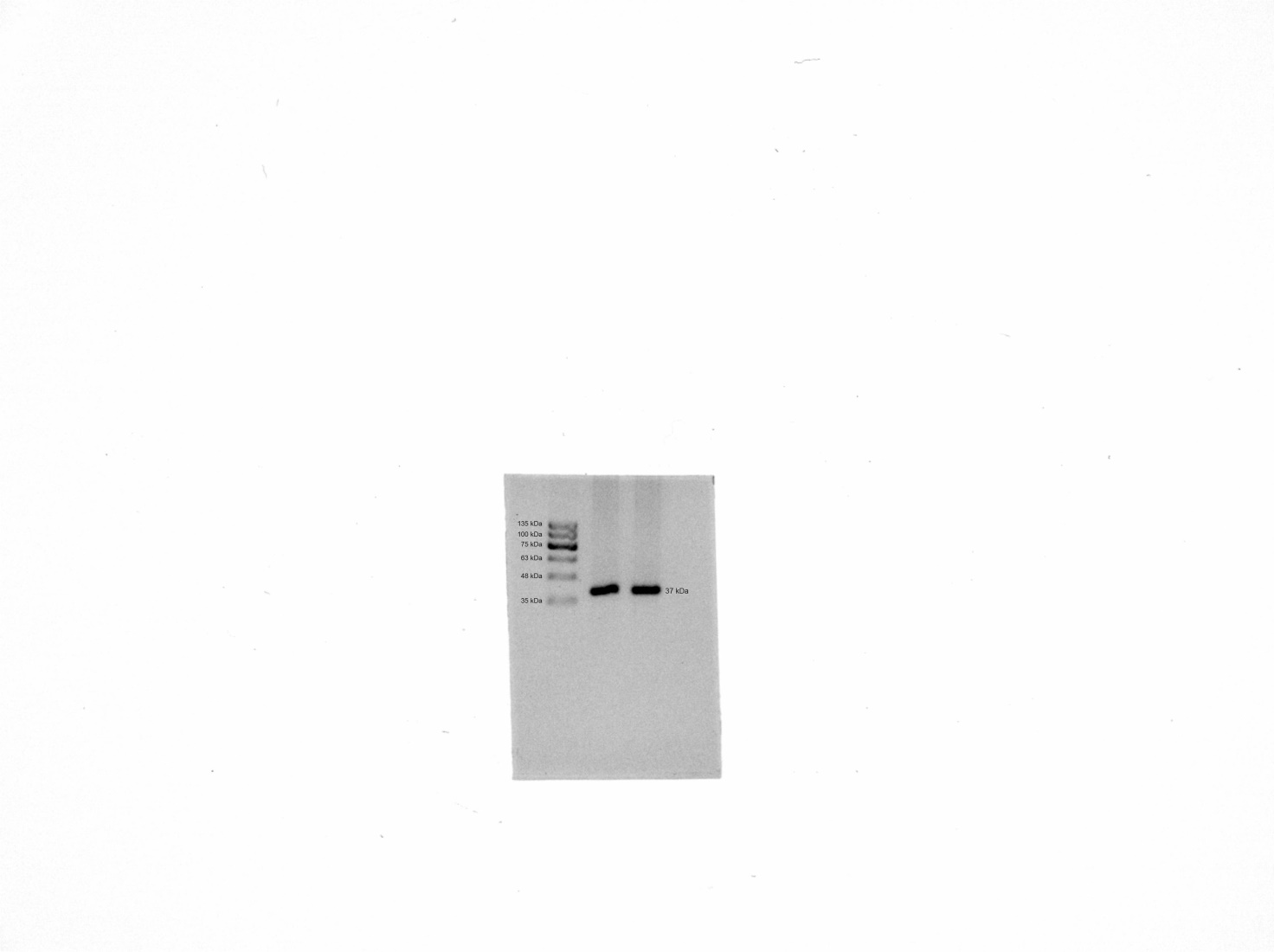


Figure3F GAPDH


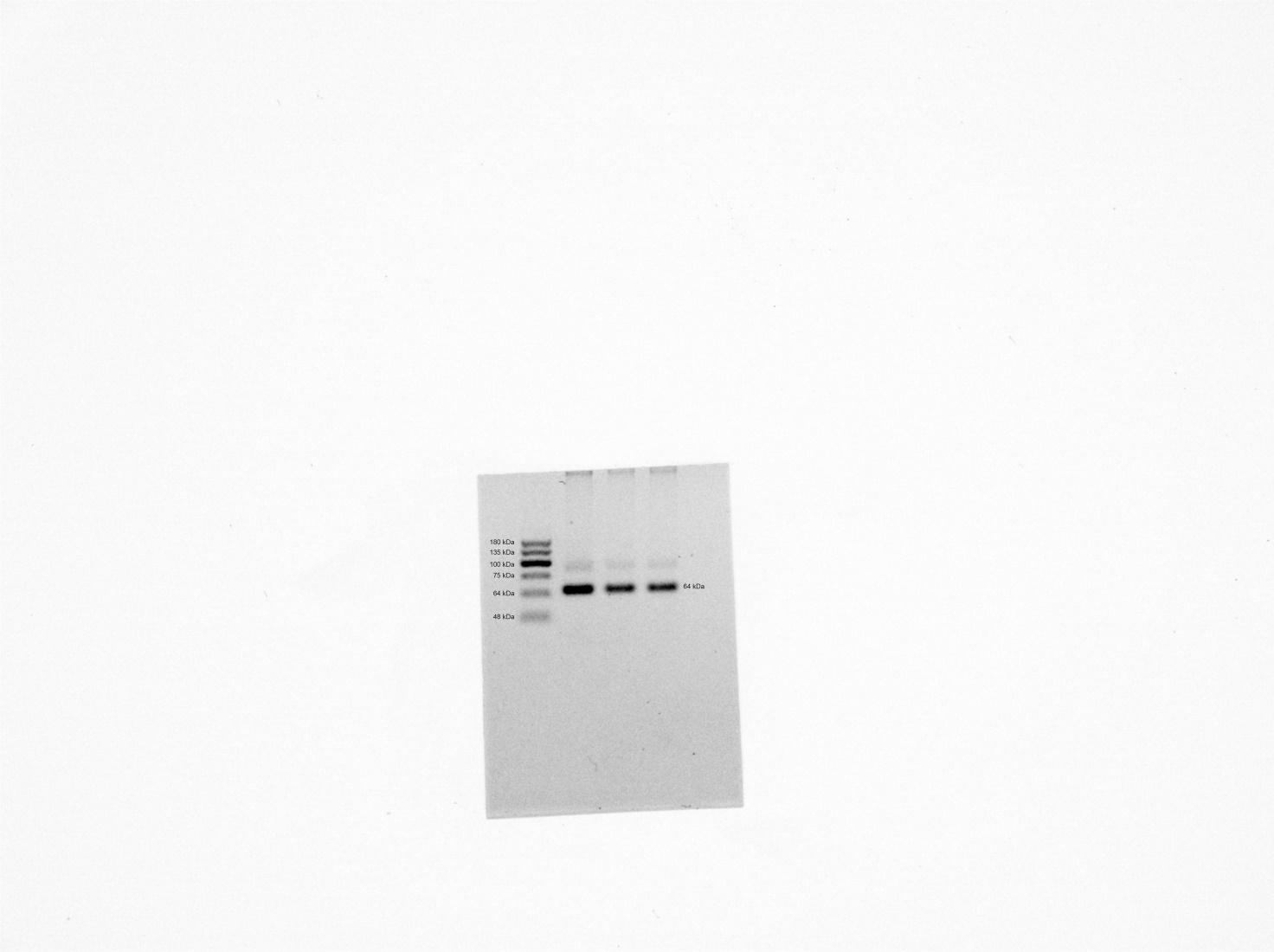


Figure4C NF-kb p65


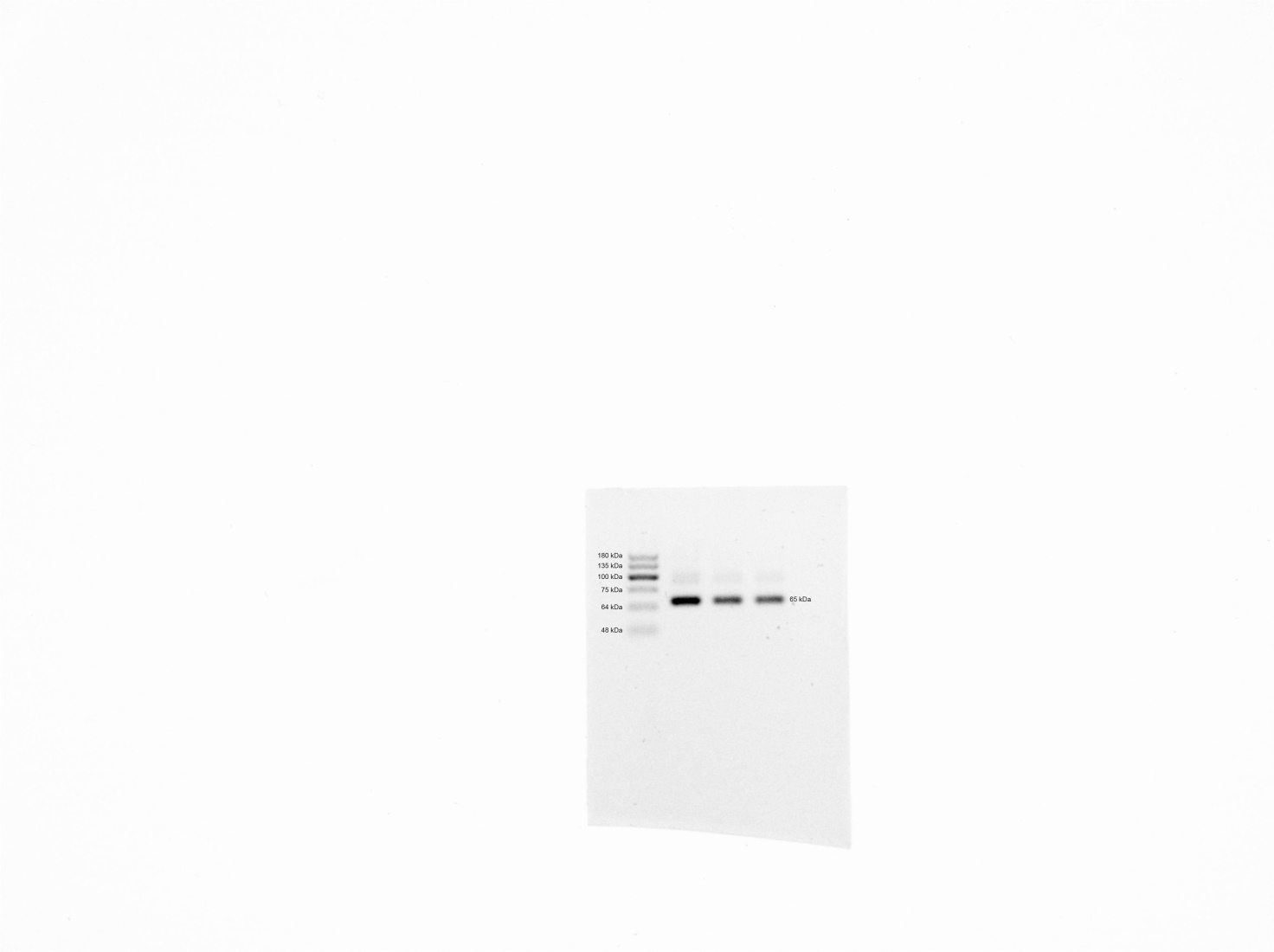


Figure4C p-NF-kb p65


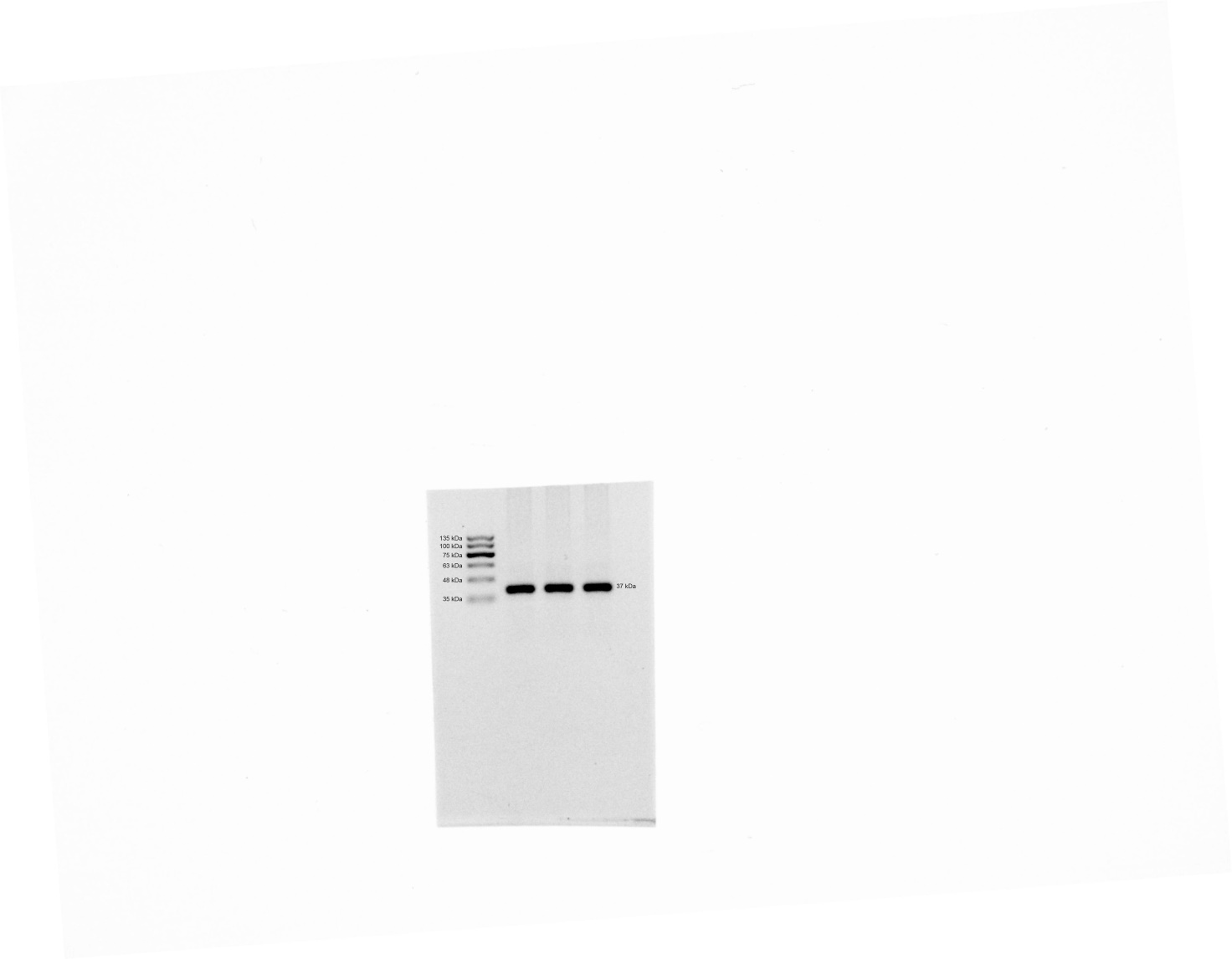


Figure4C GAPDH


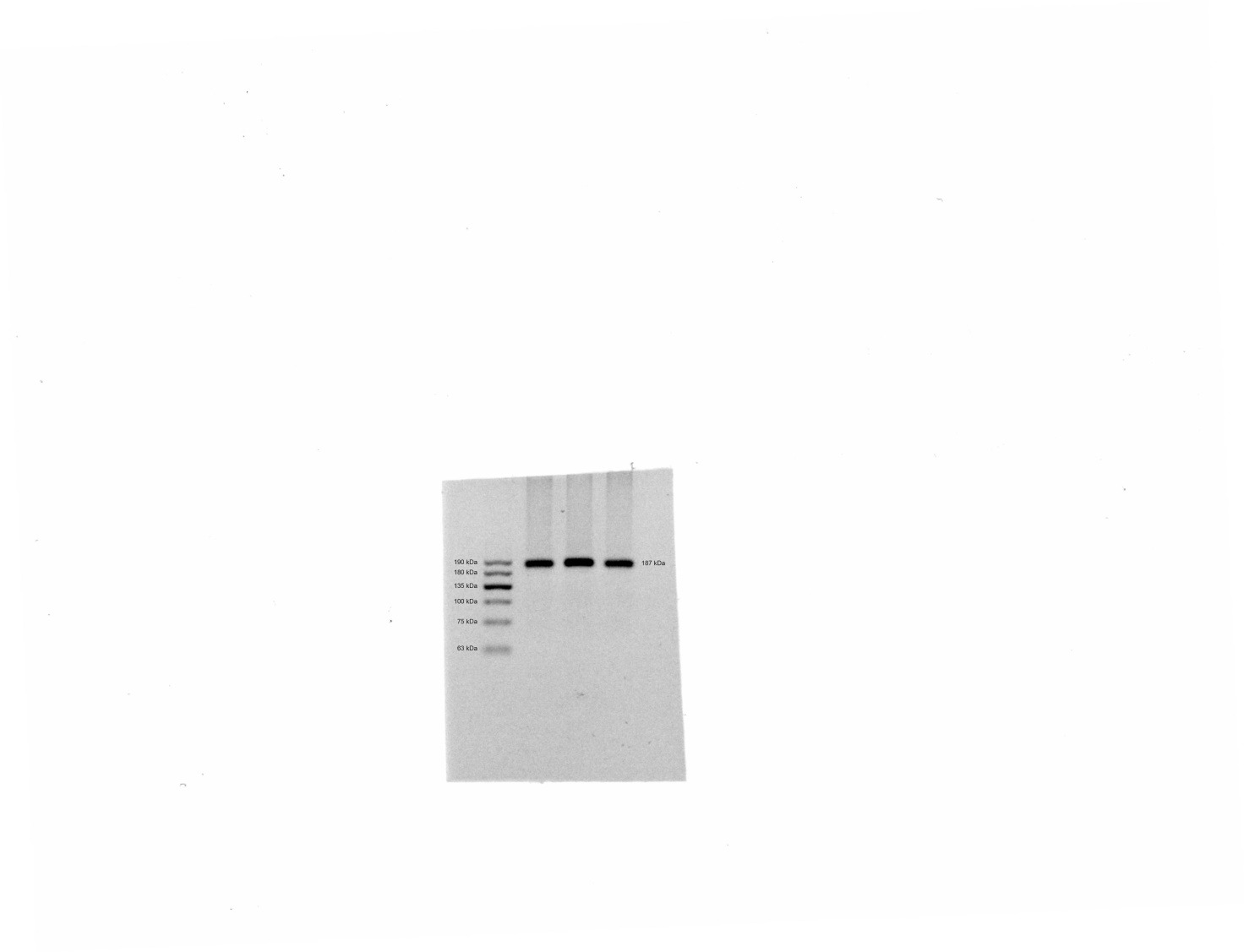


Figure4K ZO-1


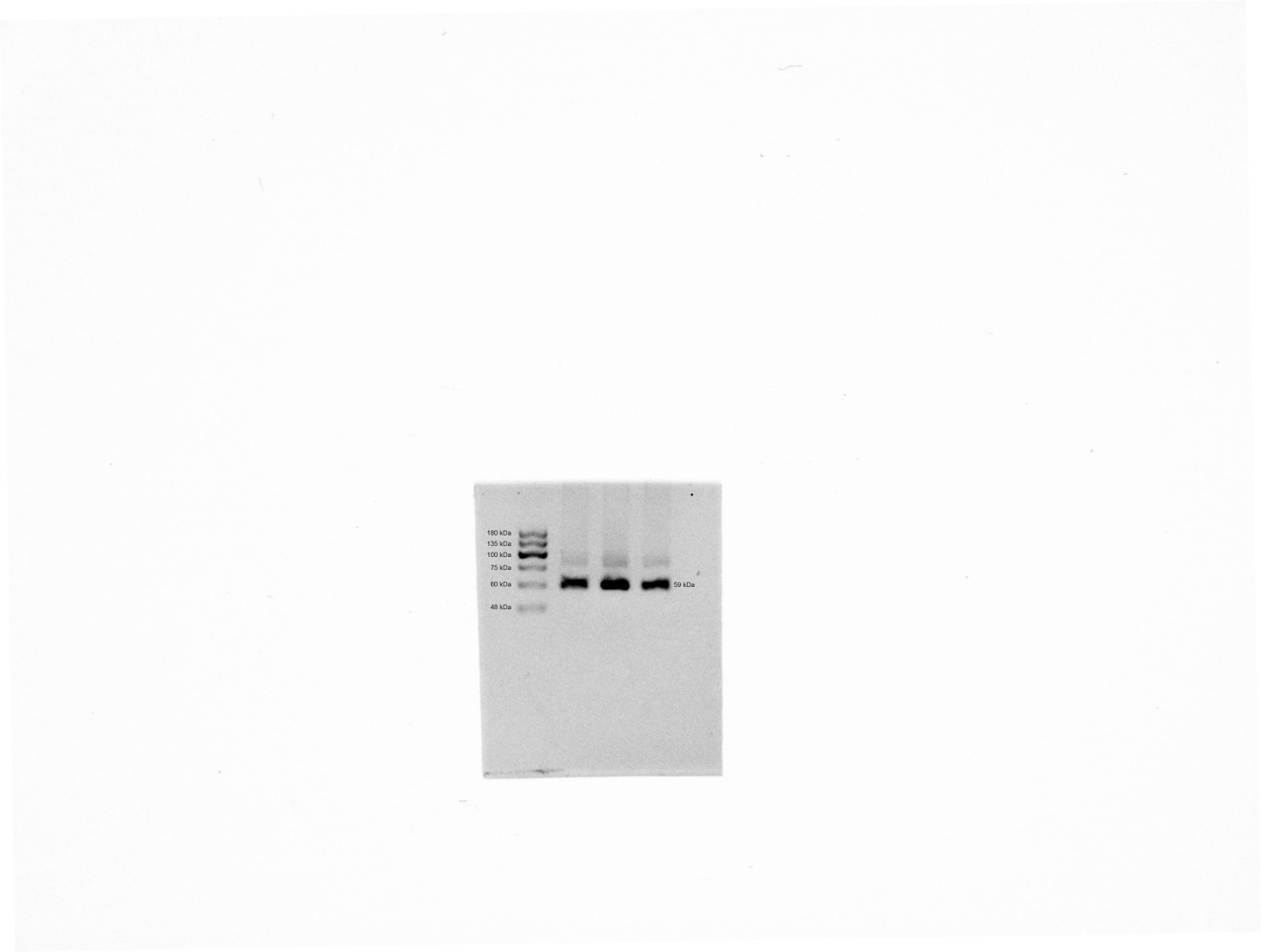


Figure4K occludin


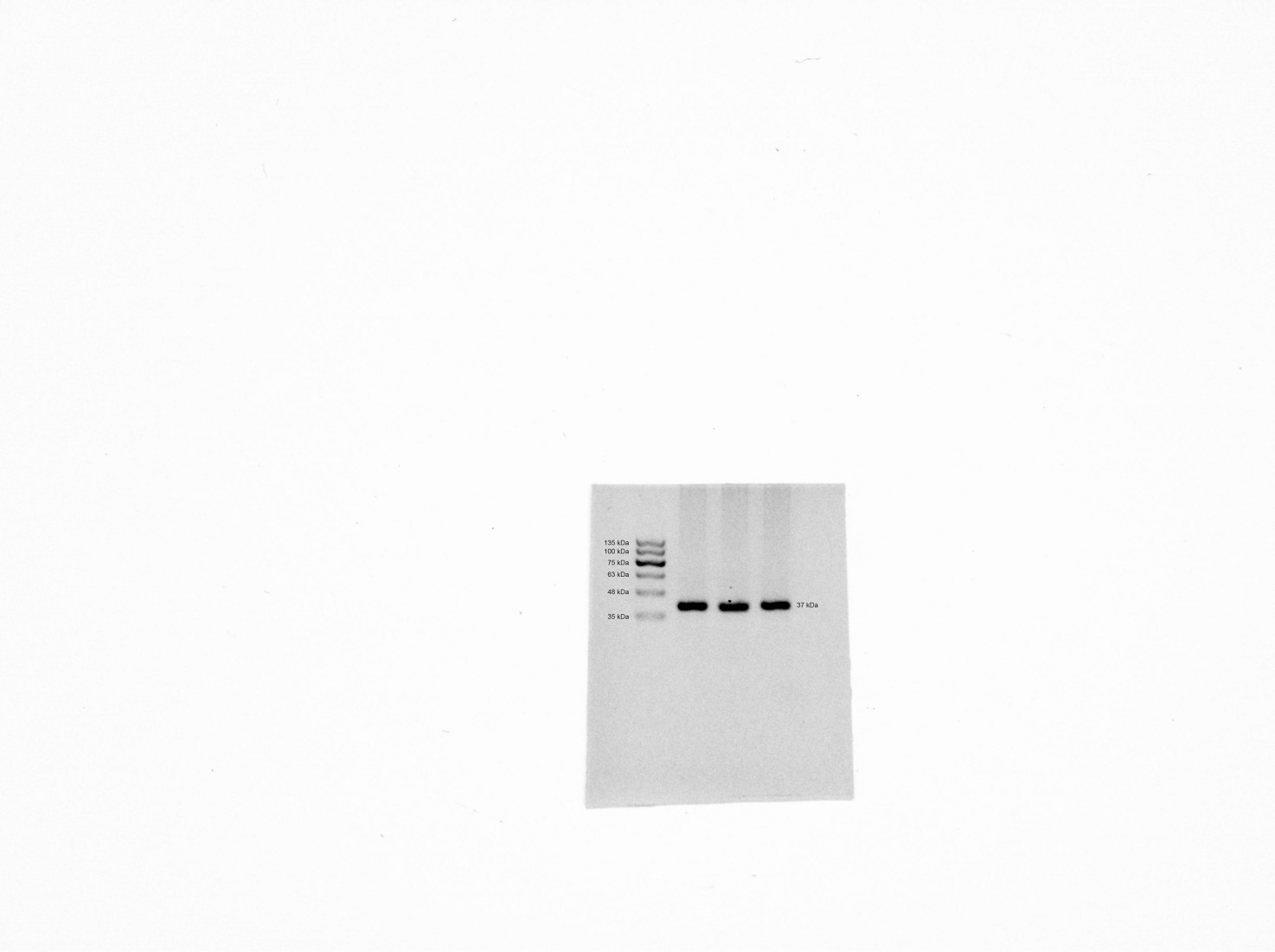


Figure4K GAPDH


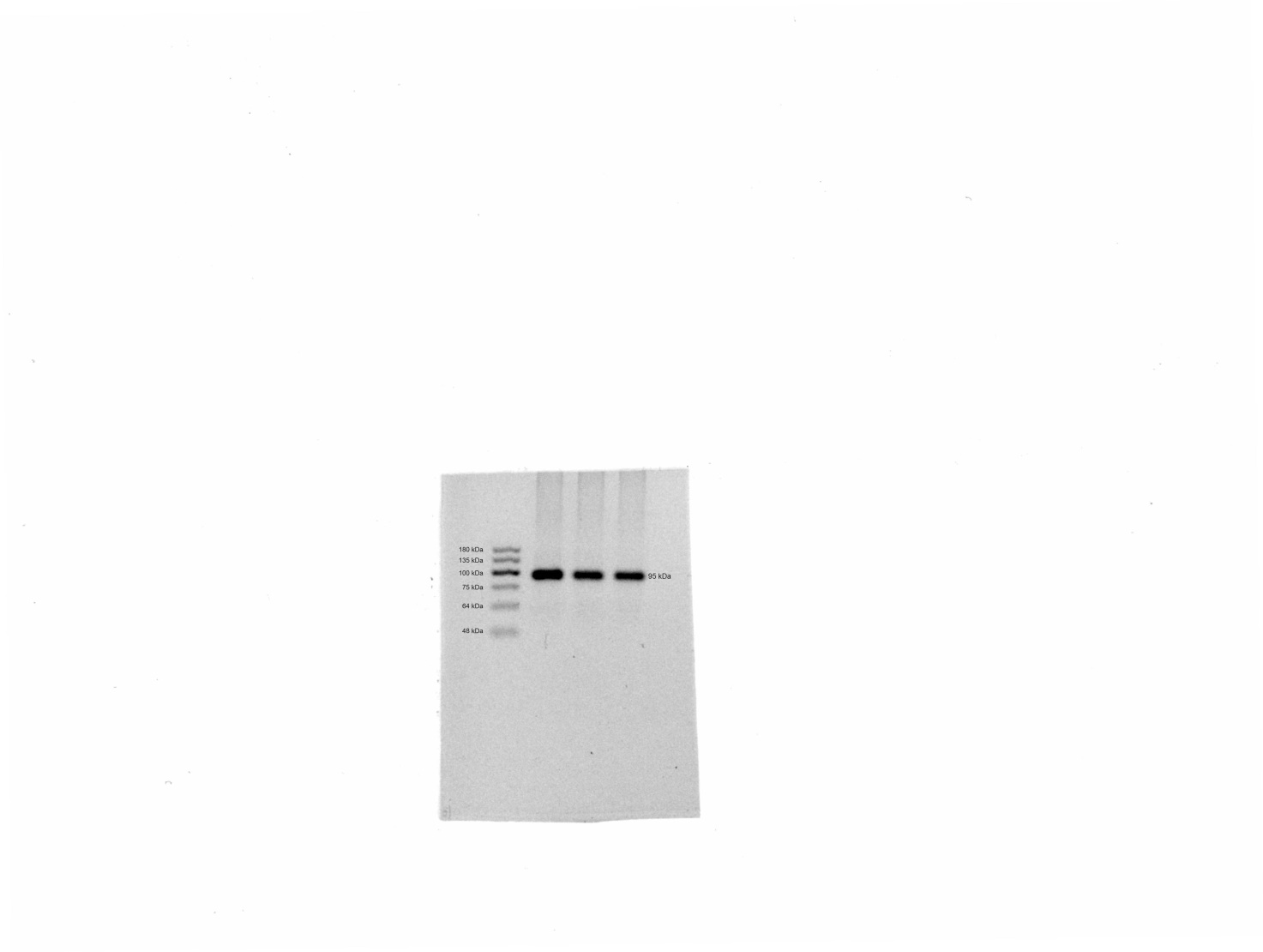


Figure5B TLR4


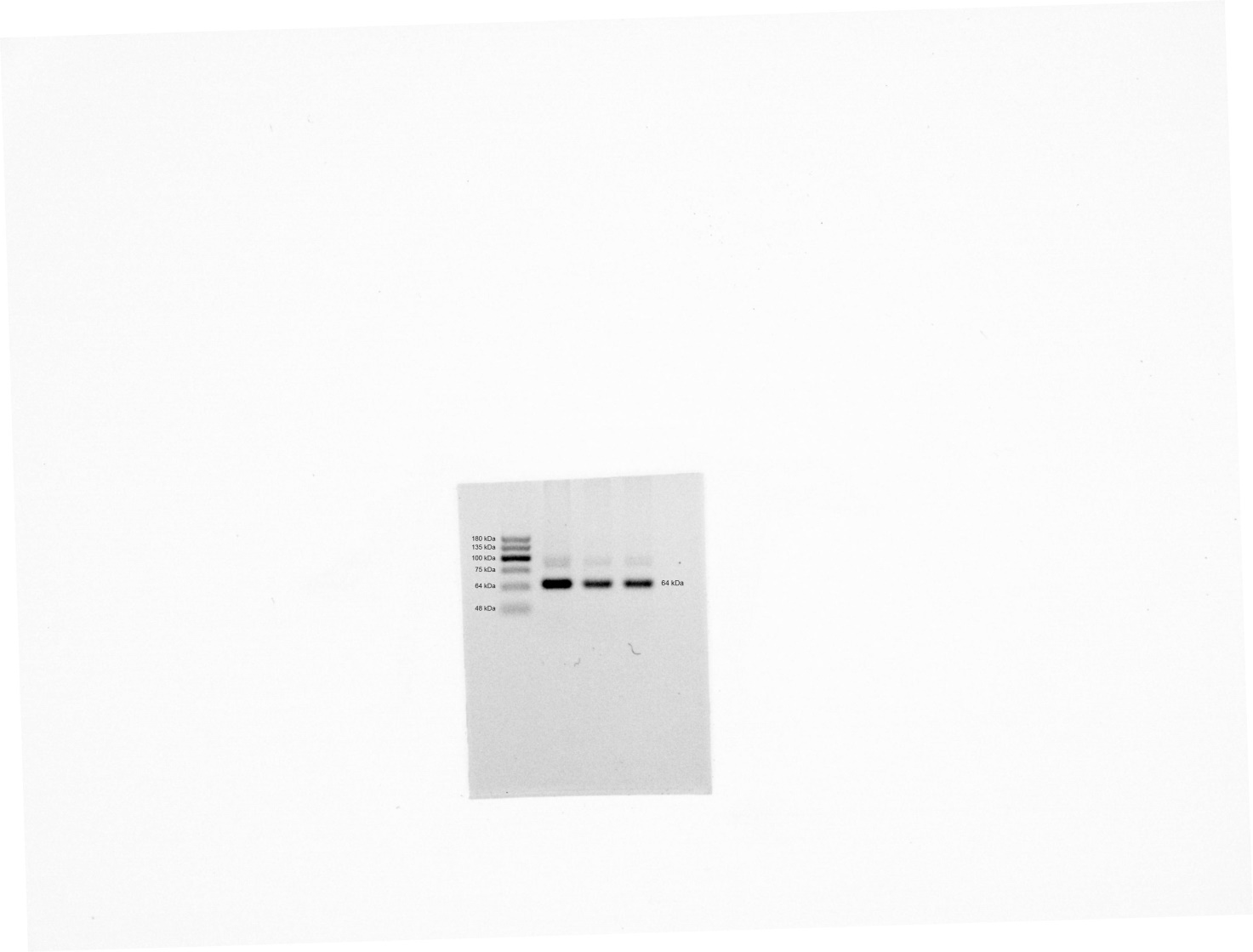


Figure5B NF-_KB_ p65


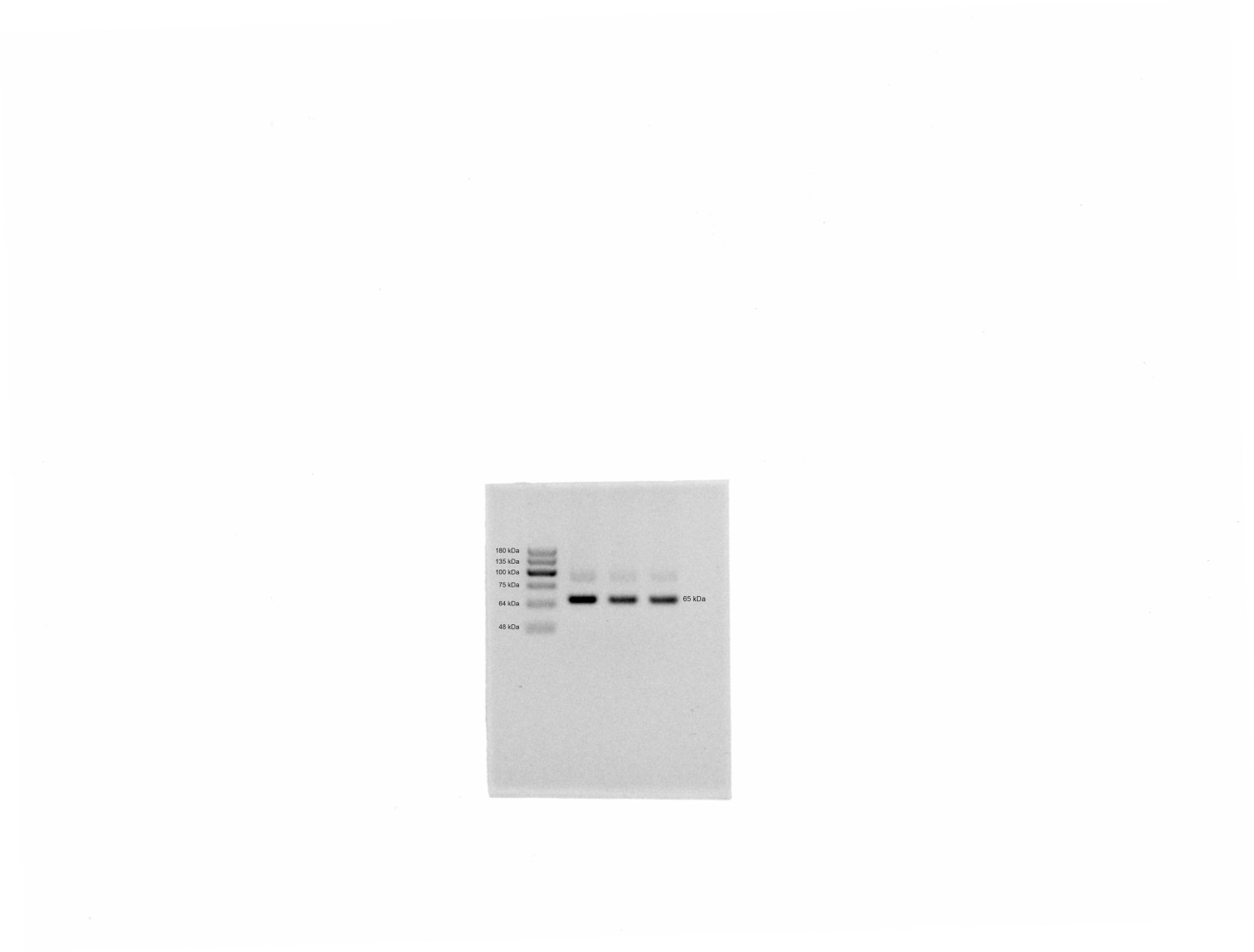


Figure5B p-NF-kb p65


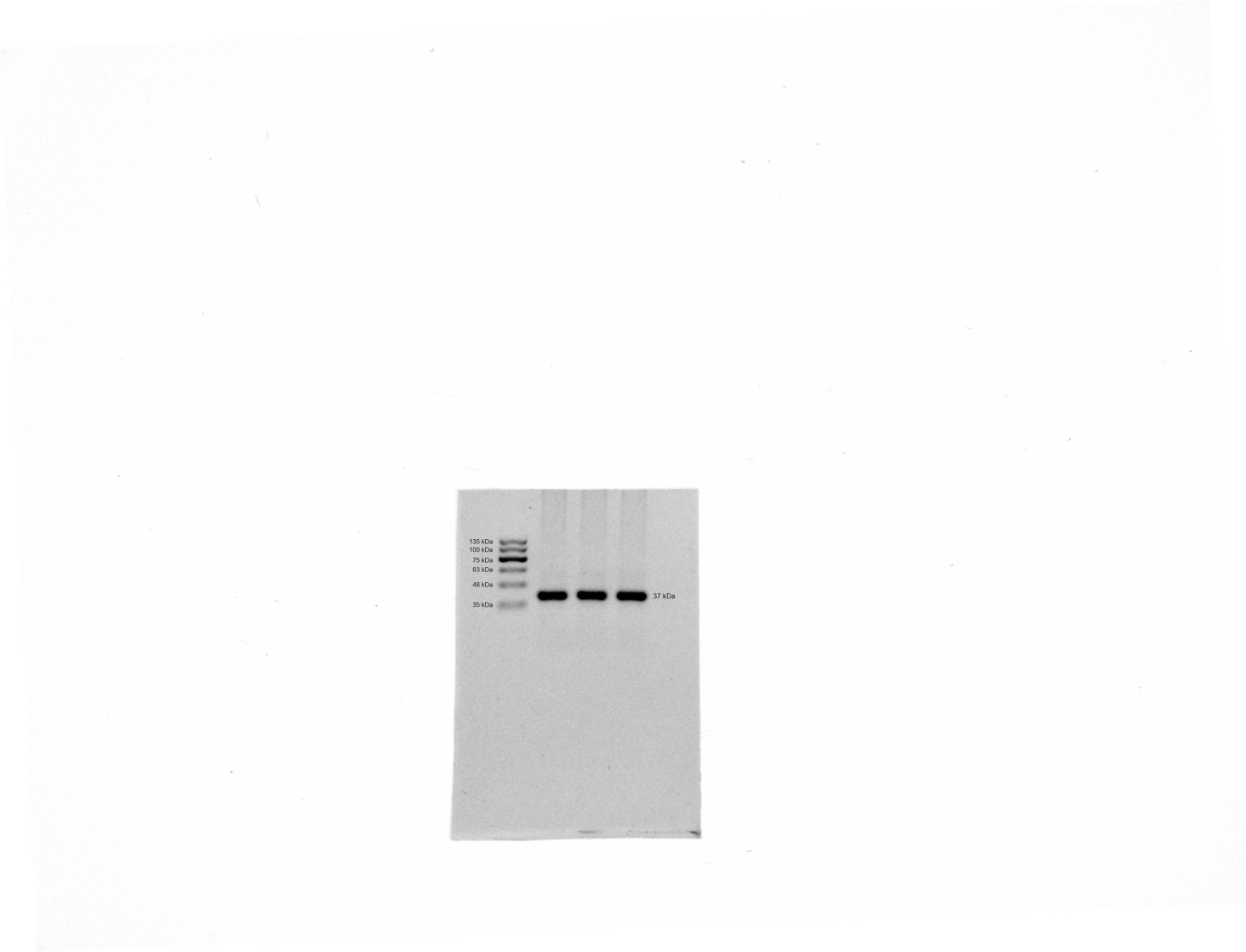


Figure5B GAPDH


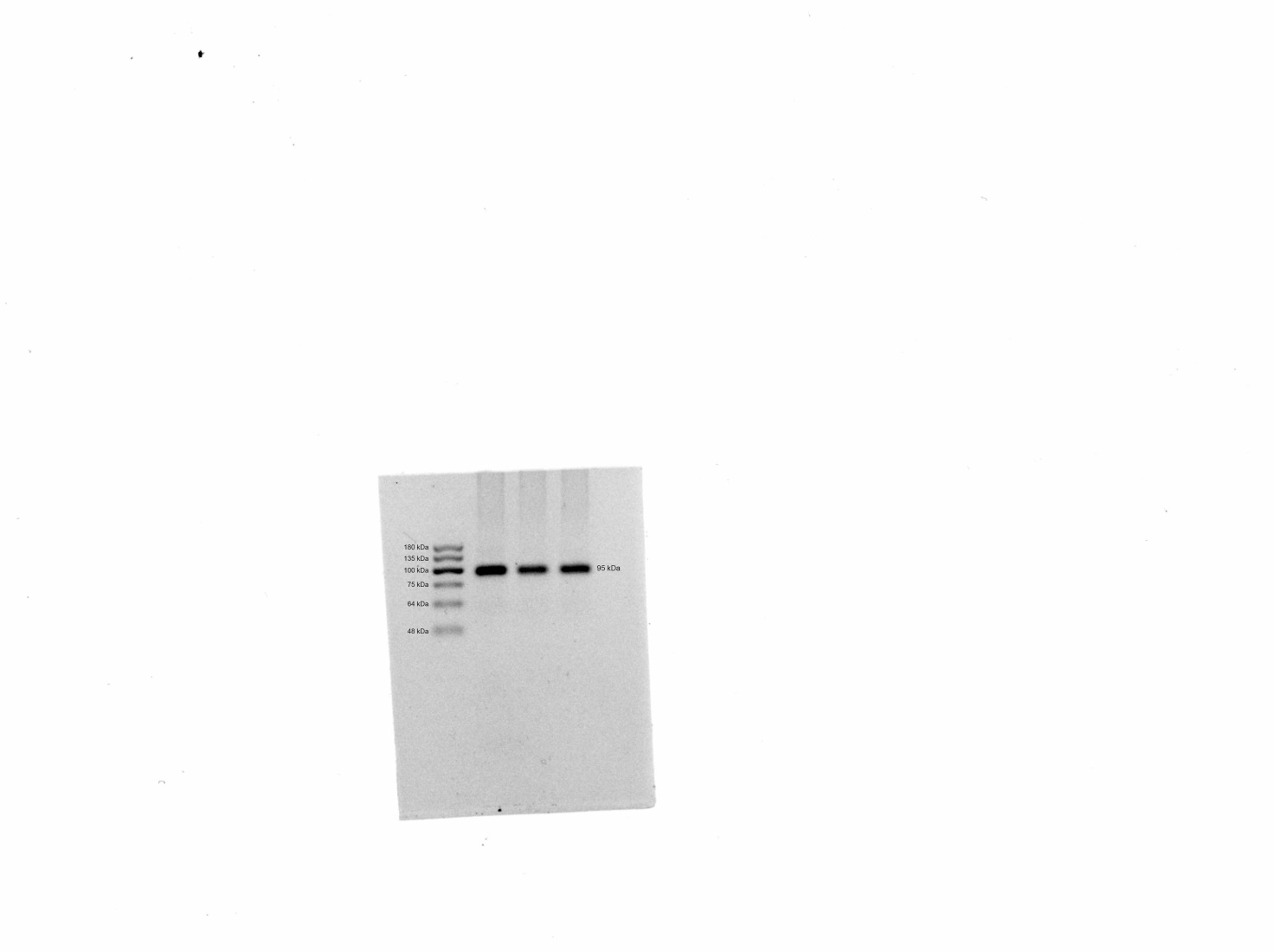


Figure6B TLR4


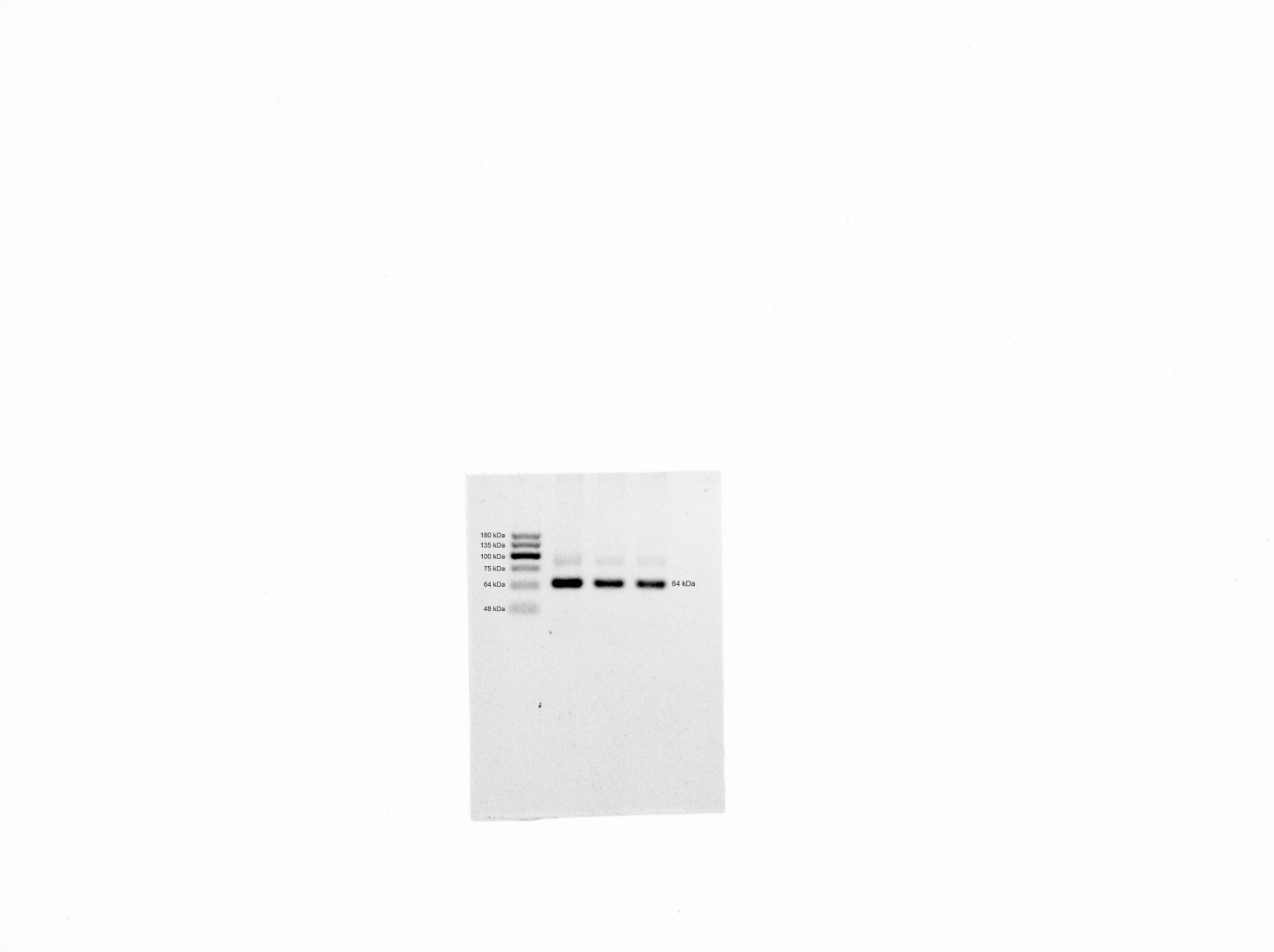


Figure6B NF-kb p65


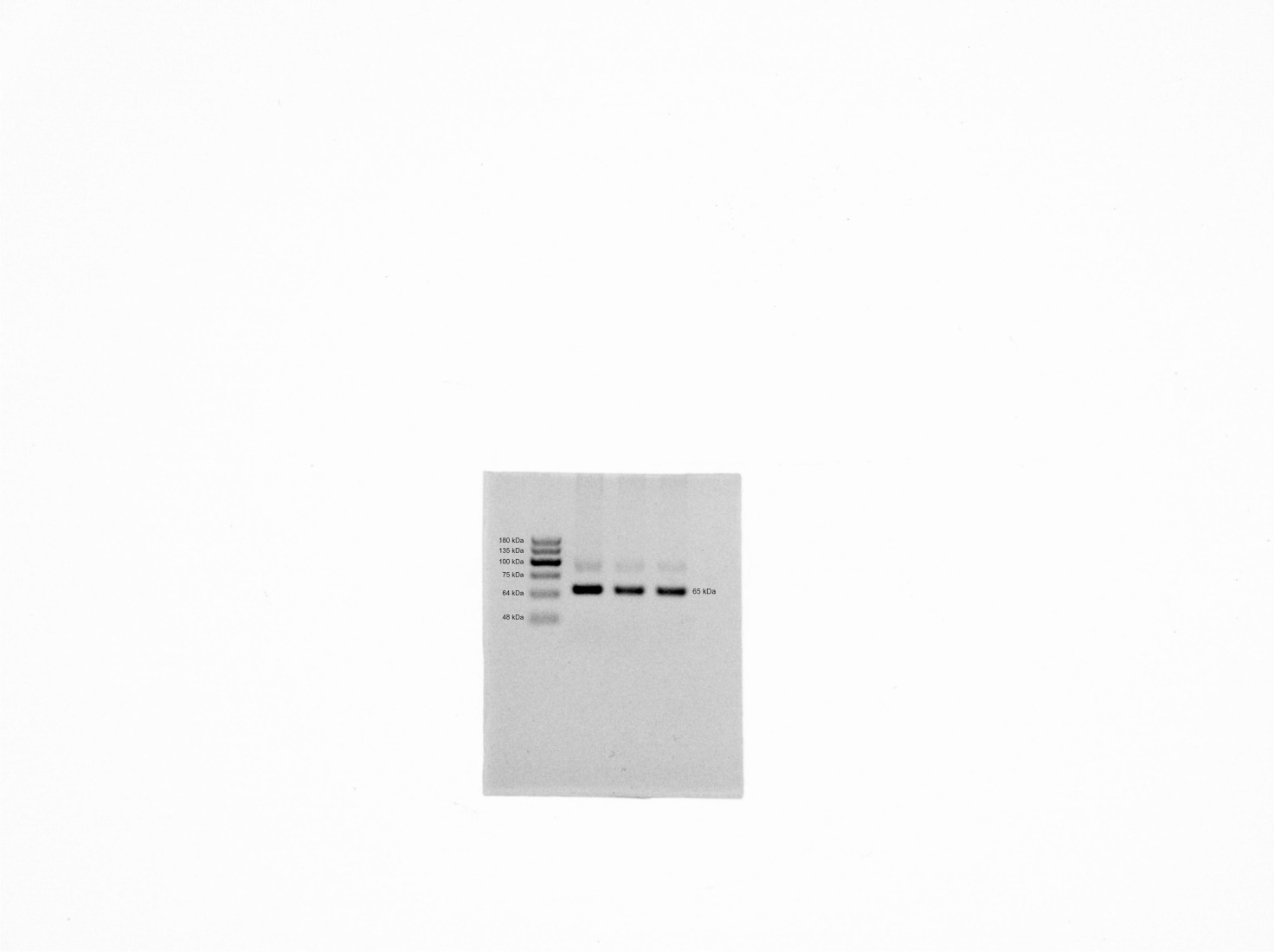


Figure6B p-NF-kb p65


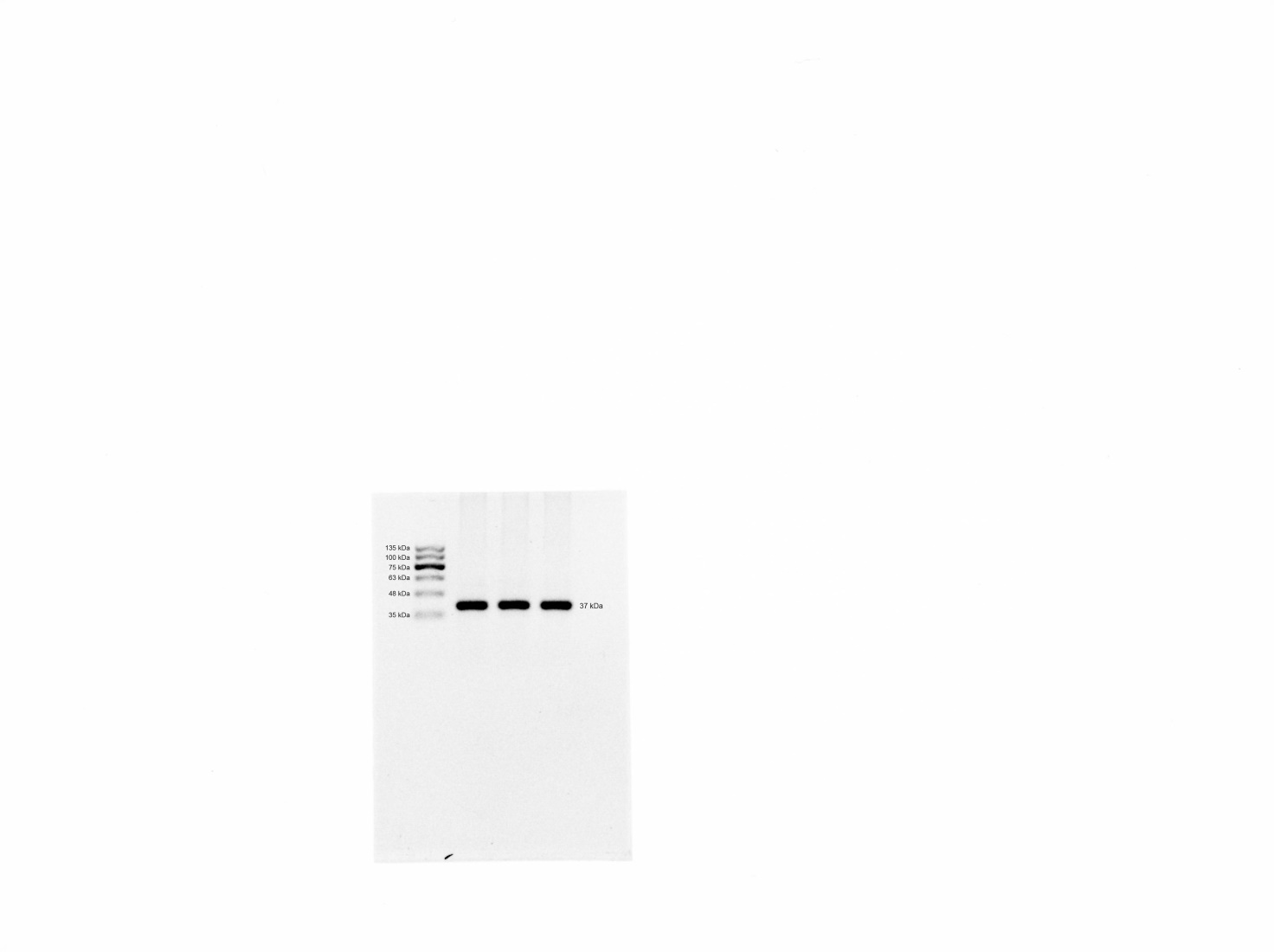


Figure6B GAPDH
